# What drives genetic and phenotypic divergence in the Red‐crowned Ant tanager (*Habia rubica*, Aves: Cardinalidae), a polytypic species?

**DOI:** 10.1002/ece3.5742

**Published:** 2019-10-21

**Authors:** Sandra M. Ramírez‐Barrera, Julián A. Velasco, Tania M. Orozco‐Téllez, Alma M. Vázquez‐López, Blanca E. Hernández‐Baños

**Affiliations:** ^1^ Posgrado en Ciencias Biológicas Universidad Nacional Autónoma de México Ciudad de México Mexico; ^2^ Departamento de Biología Evolutiva Facultad de Ciencias Museo de Zoología Universidad Nacional Autónoma de México Ciudad de México Mexico; ^3^ Centro de Ciencias de la Atmósfera Universidad Nacional Autónoma de México Ciudad de México Mexico

**Keywords:** ecological speciation, genetic structure, isolation by distance, landscape genetics, phenotypic variation, polytypic species

## Abstract

**Aim:**

The effects of geographic and environmental variables on patterns of genetic and phenotypic differentiation have been thoroughly studied. Ecological speciation involves reproductive isolation due to divergent natural selection that can result in a positive correlation between genetic divergence and adaptive phenotypic divergence (isolation by adaptation, IBA). If the phenotypic target of selection is unknown or not easily measured, environmental variation can be used as a proxy, expecting positive correlation between genetic and environmental distances, independent of geographic distances (isolation by environment, IBE). The null model is that the amount of gene flow between populations decreases as the geographic distance between them increases, and genetic divergence is due simply to the neutral effects of genetic drift (isolation by distance, IBD). However, since phenotypic differentiation in natural populations may be autocorrelated with geographic distance, it is often difficult to distinguish IBA from the neutral expectation of IBD. In this work, we test hypotheses of IBA, IBE, and IBD in the Red‐crowned Ant tanager (*Habia rubica*).

**Location:**

Mesoamerica (Mexico—Central America) and South America.

**Taxon:**

*Habia rubica* (Aves: Cardinalidae).

**Methods:**

We compiled genetic data, coloration, and morphometric data from specimens from collections in Mexico and the United States. We used the Multiple Matrix Regression with Randomization (MMRR) approach to evaluate the influence of geographic and environmental distances on genetic and phenotypic differentiation of *H. rubica* at both phylogroup and population levels.

**Results:**

Our results provide strong evidence that geographic distance is the main driver of genetic variation in *H. rubica*. We did not find evidence that climate variation is driving population differentiation in this species across a widespread geographic region.

**Main conclusions:**

Our data point to geographic isolation as the main factor structuring genetic variation within populations of *H. rubica* and suggest that climate is not playing a major role in genetic differentiation within this species.

## Graphical abstract

Our study provides strong evidence that geographic distance is the main driver of genetic variation in *Habia rubica*. Our data point to geographic isolation as the main factor structuring genetic variation within populations of *H. rubica* and suggest that climate is not playing a major role in genetic differentiation within this species.

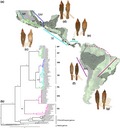

## 1 INTRODUCTION

**Figure 1:**
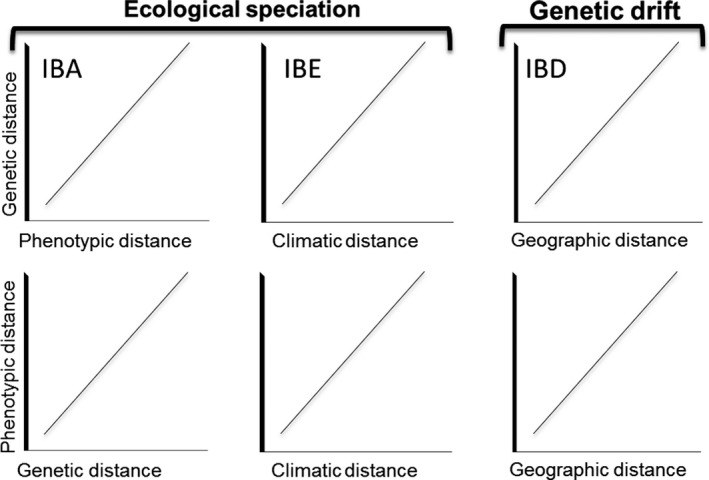
Simplified predictions of correlations between of genetic, phenotypic, climatic, and geographic distance matrices under the Isolation by adaptation, isolation by environment, and isolation by distance hypotheses. Isolation by adaptation (IBA) refers to a positive correlation between phenotypic differentiation (subject to sexual or natural selection) and genetic differentiation. This correlation occurs when the gene flow between populations is restricted by individual mate preferences or by increased mortality of immigrant phenotypes. Isolation by environment (IBE) refers to a positive effect of environmental differentiation on genetic or phenotypic differentiation, which occurs when the gene flow between populations is restricted by individual preferences to remain in a particular environment or by selection against dispersers moving between populations. Isolation by distance (IBD) refers to a positive effect of geographic separation on genetic or phenotypic differentiation as a consequence of restricted gene flow when the populations are isolated, either by geographic distances or by landscape barriers

Many animal species show considerable levels of intraspecific variation that reflect the effects of selective and/or neutral evolution (Lande, 1976; Morales et al., 2017; Nosil, 2012; Seeholzer & Brumfield, 2017; Zamudio‐Beltrán & Hernández‐Baños, 2015). Within natural populations, genetic and phenotypic divergence may be influenced by factors such as sexual and natural selection, genetic drift, and geographic isolation (Bohonak, 1999; Slatkin, 1987; Wang & Summers, 2010; Wright, 1943). Although patterns of genetic differentiation often reflect spatial variation in gene flow, the landscape itself might influence this gene flow in at least two important ways: through geographic isolation and through ecological isolation (Coyne & Orr, 2004; Thorpe, Surget‐Groba, & Johansson, 2008). Geographic isolation (Dobzhansky, 1937) proposes that geographic distances and barriers restrict gene flow among populations (Wang, 2013; Wang, Glor, & Losos, 2012), resulting in a positive correlation between genetic divergence and geographic factors (isolation by distance, IBD; Wright, 1943). Ecological isolation (Dobzhansky, 1937), on the other hand, occurs when gene flow is reduced among populations due to the effect of one or both of two different processes—isolation by adaptation and isolation by environment. Isolation by adaptation (IBA; Rundle & Nosil, 2005) is defined as the effect of environmental gradients that results in divergent natural selection that can lead to adaptive phenotypic divergence between populations, resulting in a positive correlation between genetic divergence and adaptive phenotypic differentiation (Funk, 1998; Guayasamin et al., 2017). Isolation by environment (IBE, Wang & Bradburd, 2014) is defined as the occupation of two populations in different points on the ecological gradient. This process is observed when the phenotypic target of selection is unknown or is not easily measured, and then, the environmental variation can be used as a proxy and a positive correlation between genetic divergence and environmental dissimilarity is expected. These hypotheses are not mutually exclusive; spatial genetic divergence among populations can result from reduced gene flow associated with both geographic and ecological factors (Figure 1).

**Figure 2:**
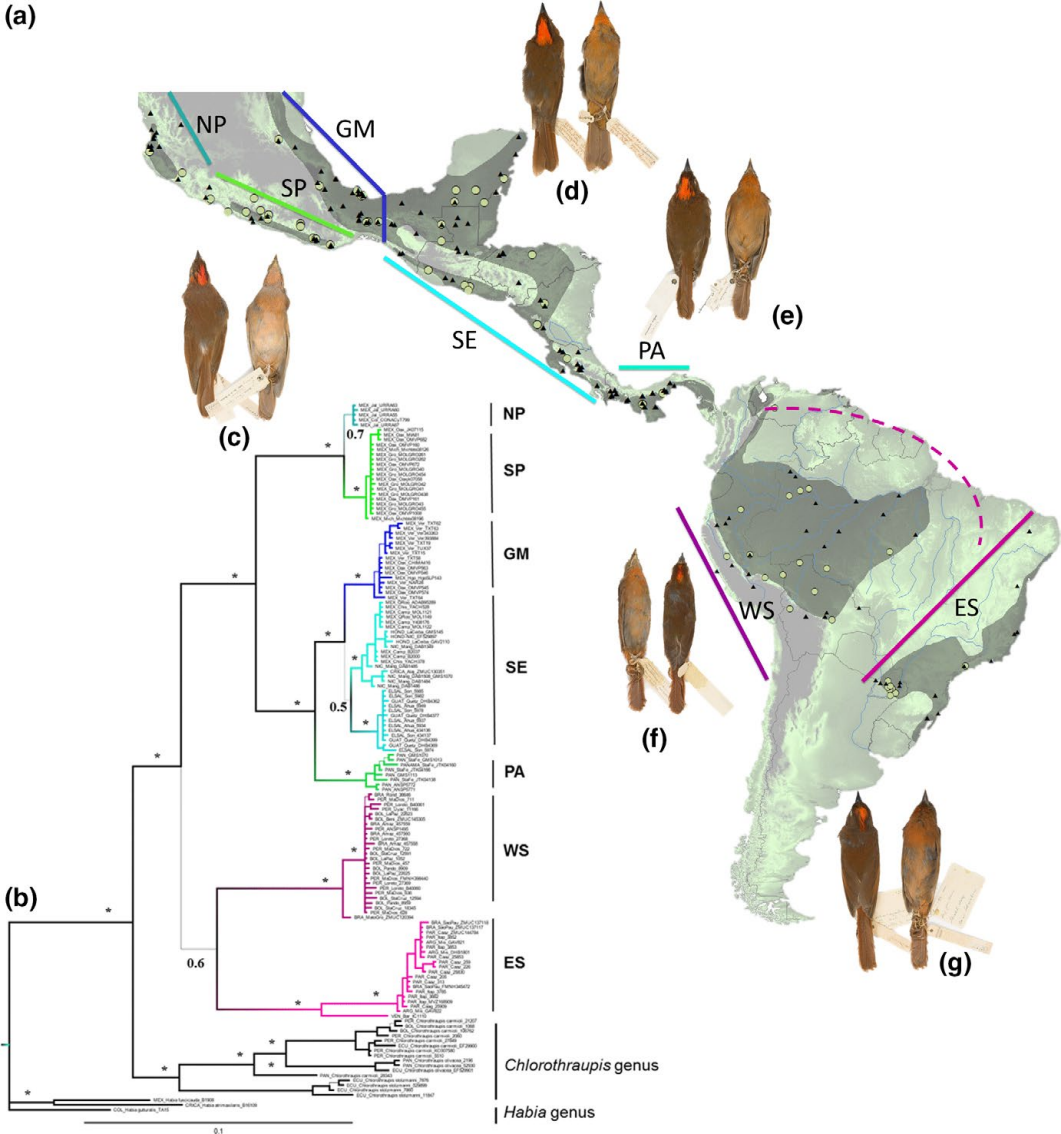
(a) Geographic distribution (gray shading) and sampling points of *Habia rubica* (green points in genetic sampling and black triangles in phenotypic sampling). (b) Phylogenetic consensus tree representing the relationship among *H. rubica* phylogroups based on Bayesian inference from a mitochondrial dataset obtained from Ramírez‐Barrera et al. (2018). The values on the branches indicate posterior probability. Both the map and the phylogenetic tree show the geographic position of the sampled phylogroups: NP, northern pacific of Mexico; SP, southern pacific of Mexico; GM, Gulf of Mexico; SE, southeastern Mexico and northern Central America; PA, Panama; WS, western South America and ES, eastern‐northwestern South America. (c–g) Color variation in plumage of phylogroups of *H. rubica* from coast of the Mexican Pacific (c); phylogroups from Gulf of Mexico to Costa Rica (d); phylogroup from Panama (e); phylogroups from western South America (f) and phylogroups from eastern‐northwestern South America (g). Photographs by Sahid M. Robles

Testing the associations between morphological, color, environmental, geographic, and genetic variation is the first step for understanding the relative contributions of these different potential drivers of genetic and phenotypic variation among populations within a species. Examining patterns of IBD and IBE is an important starting point for understanding how landscapes shape patterns of genetic variation in nature (Wang & Summers, 2010). Several factors make the Red‐crowned Ant tanager (*Habia rubica*) a good model for performing tests of IBD and IBE. It is a highly polytypic species that is distributed from central Mexico to northeastern Argentina and southeastern Brazil (Figure 2a), and it has a continental distribution that encompasses a variety of suitable environments. It also has extensive geographically structured color variation that is well documented in species descriptions (Hilty, 2011), and plumage coloration differentiation among its genetic populations has been objectively measured using reflectance spectrometry (Lavinia et al., 2015). The most recent phylogeographic study indicated that the genetic variation of this species is geographically structured into seven phylogroups, which have been proposed for elevation to the category of species (Ramírez‐Barrera, Hernández‐Baños, Jaramillo‐Correa, & Klicka, 2018). Five of these phylogroups are distributed in the Mesoamerica region (from Mexico to Panama), and two are from the western and eastern‐northwestern parts of South America (Figure 2b), and these last two previously described by Lavinia et al. (2015). Finally, the relationship between the phylogroups in this species may be determined by the action of various historical processes that have promoted deep genetic structure (Lavinia et al., 2015; Ramírez‐Barrera et al., 2018).

The *H. rubica* species complex contains up to 17 described subspecies, defined mainly by plumage color variation and geographic distribution. The geographic variation in plumage color of this species is mainly in the dorsal (from the crown to the tail) and ventral (from the throat to the lower belly) brightness. However, both hue and saturation also present some variation, ranging from pale pink in the populations from the Mexican Pacific to dark red in populations from eastern South America (Hilty, 2011). Populations from the eastern of Mexico to the Amazon are intermediate, with hues ranging from brown to brick red to salmon (Figure 2c–g; Hilty, 2011).

In this study, we use a multivariate approach to disentangle the relative influence of geographic and environmental distances on genetic and phenotypic differentiation of populations across the range of *H. rubica*. We test the IBA, IBE, and IBD hypotheses on the genetic structuring of the previously identified phylogroups (Lavinia et al., 2015; Ramírez‐Barrera et al., 2018; see Figure 1c) and among populations across the entire distribution of this species. We estimated the “relative importance” of each predictor using standardized regression coefficients from MMRR (Multiple Matrix Regression with Randomization) analysis. We expected genetic divergence to be positively correlated with phenotypic divergence under IBA, environmental divergence under IBE, and/or geographic distance under IBD (Figure 1).

## 2 MATERIALS AND METHODS

### 2.1 Genetic data

Our genetic sampling for this work comprised 124 mitochondrial DNA sequences (ND2 gene, ~1,041 bp) from *H. rubica* from a recent study (Ramírez‐Barrera et al., 2018). This sample covers most of the geographic range of *H. rubica* and can therefore be considered a relatively good proxy for the total genetic diversity of the populations of this species (Figure 2a).

We generated two matrices of genetic distances using these molecular data. The first (124 sequences) was based on the affiliation to a given phylogroup as defined in Ramírez‐Barrera et al. (2018; see Figure 2b), using the following groups: Mexican Northern Pacific (NP), Mexican Southern Pacific (SP), Gulf of Mexico (GM), southeastern Mexico and northern Central America (SE); Panama (PA); western South America (WS), and eastern/northwestern South America (ES, the northwest population is represented by a single sample from Venezuela). We used the program MEGA v7 (Kumar, Stecher, & Tamura, 2016) to generate this matrix, grouping individuals by phylogroup. The second matrix was generated using all possible pairs of individuals for which it was possible to match genetic and phenotypic data (110 males and 104 females, see “Data Matching” section below). Some sequences were used both in the database of males and females, and for this reason, a total database of 214 individuals were obtained from a genetic database of 124 sequences. This distinction between matrices allows us to identify the strength of the correlation between pairs of variables, so that if we obtained similar results, we could affirm that environmental variation is an important factor that influences the differentiation between populations on a continental scale. The data processing needed for this estimation was carried out using the *phyDat*, *modelTest*, and *dist.ml* functions of the “phangorn” package in R v3. 5. 0 (Schliep, 2011; R Foundation for Statistical Computing, Vienna, Austria). The Jukes–Cantor model was the nucleotide substitution model that best fit the data (Jukes & Cantor, 1969).

### 2.2 Morphometric and color data

We obtained morphometric and color data for 339 adult specimens of *H. rubica* (see Appendix S1: Table S1.1). Our phenotypic sampling of *H. rubica* was conducted at the level of phylogroups including different numbers of females (NP = 8, SP = 17, GM = 27 SE = 32, PA = 15, WS = 28, ES = 20; total = 147) and males (NP = 10, SP = 15, GM = 32, SE = 59, PA = 12, WS = 33, ES = 31; total = 192), (Appendix S2: Figure S2.1). The morphometric and color data obtained were from specimens deposited in the following collections: Museo de Zoología “Alfonso L. Herrera,” UNAM, Mexico (MZFC); Colección Nacional de Aves, UNAM, Mexico (CNAV‐IB); the Ornithological Collection of the American Museum of Natural History, New York (AMNH), and the Ornithological Collection of the Smithsonian Institution, Washington D. C (SI). Sexual maturity was corroborated from collection data. The distribution of this sample covers the majority of the geographic and environmental range of the species (Figure 2a).

For each specimen, we recorded wing length, tarsus length, and tail length using a Mitutoyo digital caliper with 0.01 mm accuracy, taking the average of three independent repetitions of each measurement per individual for use in subsequent analyses. Prior to the main analyses, we tested whether there was sexual dimorphism in the morphometric measurements using *t* tests and corroborated the degree of within‐individual correlation between variables using *cor* function in R. We conducted a principal component analysis—PCA—of these three morphometric variables and extracted the scores of the first principal component (PC1) as a proxy for body size (see Seeholzer & Brumfield, 2017 for a similar approach). PC1 explained 73% of the variation in body size among males and 75% among females in the phylogroup‐level analysis and 66% among males and 64% among females in the individual‐level analysis. We tested the relationship between PC1 and latitude to explore possible latitudinal trends in body size. Finally, we converted these body size values to a distance matrix using the *dist* function in R.

We obtained plumage reflectance spectra for the following nine plumage patches: crown, upper back, lower back, rump, tail, throat, breast, upper belly, and lower belly. We quantified plumage coloration for all specimens using a USB2000 spectrophotometer (Ocean Optics) with an Ocean Optics PX‐2 pulsed xenon light source, connected to a bifurcated fiber‐optic probe. The probe was fitted with a rubber stopper to exclude ambient light and maintain a constant distance and 90° angle between the probe tip and the plumage. Measurements were taken following standard procedures (Eaton, 2005) to record plumage reflectance for each wavelength within the avian visual spectrum, from 300 to 700 nm. We used Ocean Optics software to integrate the spectrophotometer data.

We analyzed reflectance spectra using Goldsmith's (1990) tetrahedral color space (Stoddard & Prum, 2008). This method quantifies color based on avian visual perception to be able to obtain a measure of total coloration, considering all the patches. We plotted all reflectance spectra in the avian tetrahedral color space (Stoddard & Prum, 2008), which represents the possible avian color space based on relative stimulations of the four retinal cone types. We processed the raw reflectance spectra using the “pavo” R package (Maia, Eliason, Bitton, Doucet, & Shawkey, 2013). We used the *visual model* function to determine the relative stimulation levels of the four avian cones using the *Sturnus vulgaris* (Common starling) visual model. The Common starling is the closest relative of *H. rubica* for which a spectral sensitivity function was available, and however, it is unlikely that changing the species on which the visual model is based would affect our analysis because the sensitivities of the avian cones are highly conserved (Hart, 2001). We converted the cone stimulation values (*u*,* s*,* m*,* l*) into a vector of three angles, which locates the color in the avian tetrahedral color space. We obtained three main measurements as a result of this processing: (a) the total volume occupied by the points across all body patches (color volume), (b) the mean of the hue span, and (c) mean saturation (chroma). The chroma measurement was included to avoid the underestimation of color variation in uniformly colorful birds (see Friedman & Remeš, 2017 for a similar approach).

Three distance matrices were generated from the color measurements (volume, hue, and chroma) using the *dist* function implemented in R for each phylogroup, specimen, and sex. The first included all nine color patches, the second used only the dorsal patches (crown, upper back, lower back, rump, and tail patches), and the third used only the ventral patches (throat, breast, high belly, and low belly). We tested the relationships between hue span and latitude to explore the possibility of latitudinal color trends.

### 2.3 Data matching

We used genetic and phenotypic data from the same individual (110 males, 104 females) whenever possible. When the two types of data were not available for the same individual, we matched phenotypic data to the genetic data from the closest individual available in terms of geographic proximity and membership in the same phylogroup. This association was conducted based on the georeferenced collection location of each sample (i.e., for each genetic and phenotypic sampled individual). Finally, since *H. rubica* is a species with evident sexual dimorphism in coloration and we found significant differences in body size between males and females (*p* < .01, Appendix S2: Table S2.1) genetic associations with morphometric, and color data were constructed separately for each sex. A list of full data associations is found in Appendix S1: Table S1.1.

### 2.4 Geographic and climate data

We estimated geographic and climatic distances between pairs of phylogroups and individuals. For the geographic data, we assigned each individual to its respective phylogroup, and then, we estimated a minimum convex polygon from the georeferences of each genetic and phenotypic sample obtained of each phylogroup (110 males and 104 females), and estimated the geographic centroid for each group (see Appendix S2: Figure S2.2). We conducted this analysis using ArcGIS software (ArcMAP 10.2.2). Finally, we calculated a Euclidean distance matrix in meters among all phylogroups and all individuals using the *distm* function from “geosphere” in R (Hijmans, 2014). For the climate data, we follow these steps: 1. using the same minimum convex polygon from the geographic distances analysis; 2. then, a raster file of each polygon was obtained for each polygon (phylogroup) with a resolution of 2.5′ (compatible with the resolution of the database consulted in WorldClim); 3. we obtained the coordinates of each cell (raster) in ArcGIS software (ArcMAP 10.2.2); 4. we extracted data for 19 bioclimatic variables (Appendix S2: Table S2.2) from the WorldClim database (Hijmans, Cameron, Parra, Jones, & Jarvis, 2005) for all the coordinates that make up each polygon (the number of cells varied according to the size of each polygon); and 4. the mean and median values of each bioclimatic variable were estimated; 5. both values were compared using a graph, to verify they are very similar to each other, and therefore, it is possible to use the average value as a measure of central tendency of each variable (these graphs were incorporated into the Appendix S2: Figure S2.3 and S2.4). The average value of each variable was used to estimate the environmental dissimilarity matrices between each pair of polygons using the *dist* function in R.

### 2.5 MMRR method

We used a MMRR approach to estimate the independent effects of environment and geography on genetic and phenotypic variation (Wang, 2013). This approximation is a similar to Mantel and partial Mantel test, but is extended to incorporate multiple regressions, can be extended to any numbers of variables that can be represented as distance matrices, and provides output in the form of a multiple regression equation (Wang, 2013). Thus, multiple regression analysis can estimate how a dependent variable changes with respect to multiple independent variables. A multiple regression equation for distance matrices can be estimated using standard multiple regression technique, with the exception that tests of significance must be performed using a randomized permutation because of the nonindependence of elements (Smouse, Long, & Sokal, 1986; Wang, 2013). Thus, MMRR analysis can quantify how genetic or phenotypic distances respond to changes in geographic and environmental distances (*β* = regression coefficients), the overall fit of the model (*R* ^2^ = coefficient of determination), and the significance of each variable (*p*‐values). We used the *MRM* function implemented in the R package “ecodist” (Goslee & Urban, 2007) using 1,000 permutations of the genetic, geographic, environmental, color, and morphometric distance matrices. Before the analysis, we scaled and centered (mean = 0 and *SD* = 1) all distance matrices using the *scale* function in R to obtain comparable standardized linear regression coefficients.

To explore the relative importance of geographic and environmental distances as predictors of genetic and phenotypic (i.e., body size and plumage coloration) divergence, we constructed both a multivariate model and univariate models. In each model, the geographic and environmental distance were the linear predictors of the pairwise genetic or phenotypic difference between phylogroups or individuals.

Additionally, a second analysis was performed using a smaller database composed of individuals for whom it was possible to obtain both genetic and phenotypic data. The objective of including this second analysis was to be able to compare the effect that the assignment of individuals (without their own genetic data) could have on the phylogroup that, according to its distribution, belongs. Therefore, this analysis is limited to the distribution of *H. rubica* in Mexico (see Appendix S3: Table S3.6 and S3.7).

## 3 RESULTS

### 3.1 Genetic sampling

The corrected pairwise genetic distances (expressed in percentages) between phylogroups (124 sequences) and individuals (110 males, 104 females) ranged from 1% to 7%, showing a clear signal of geographic population structure. In the pairwise comparisons by phylogroup, the largest genetic distances were between the South American phylogroups and those distributed from Panama to Mexico. The Panama phylogroup had the smallest genetic distance from the Northern Central America and Gulf of Mexico phylogroups. In addition, we observed that phylogroups from the Northern Mexican Pacific and Southern Mexican Pacific exhibited the shortest genetic distances and were most closely related with the Gulf of Mexico and Southeastern Mexico phylogroups (Appendix S3: Table S3.1).

### 3.2 Morphometric and color data

Within‐individual correlation coefficients for each pair of morphometric measures ranged between 0.2 and 0.6 (Appendix S3: Table S3.2, Figure S3.1 and S3.2). The tarsus and tail measurements had the highest PC1 weights in both sexes (Appendix S3: Table S3.3 and S3.4, Figure S3.3). With respect to latitudinal trends, latitude correlated positively with both plumage hue and body size in both sexes, and though in all cases, the coefficient of determination was rather low (hue: *R* ^2^ = 0.10 and 0.06, body size: *R* ^2^ = 0.05 and 0.07 for males and females, respectively). See Appendix S3: Table S3.5 and Figure S3.4.

### 3.3 MMR method for univariate analysis

**Figure 3:**
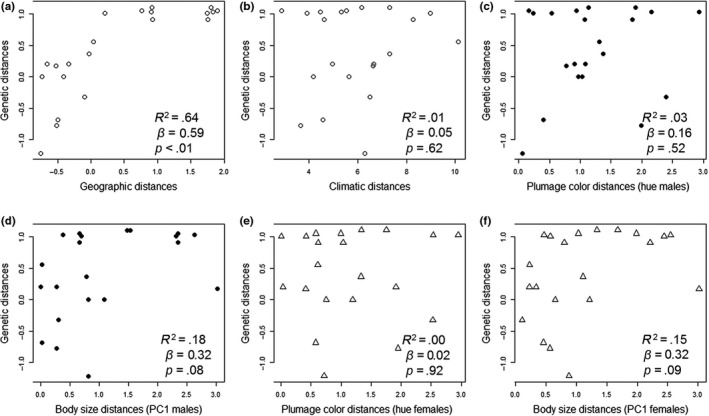
Pairwise distance matrices of mitochondrial DNA (mtDNA) against geographic, climatic, plumage color (hue), and body size distances for data grouping by phylogroups of *Habia rubica*. (a and b) geographic distances obtained with geographic centroids and environmental dissimilarity mean obtained from the coordinates per cell of the estimated raster polygon for each phylogroup (including males and females, hollow circles); (c and d) plumage color distances and body size from males (filled circles); (e and f) plumage color distances and body size from females (hollow triangles). Coefficient of determination (*R* ^2^), beta weights (*β*), and *p*‐values (*p*) of each relationship tested are shown on the graph

The results of univariate MMRR analyses showed that geography was the best predictor of genetic distance in *H. rubica* (*R* ^2^ = 0.7, Figure 3 and Table 1a). The contribution of this variable was slightly stronger in analyses performed on individual data for males and females (*β* = 0.8), than when data were grouped by phylogroup (*β* = 0.6). Climate and body size were significant only for the individual data, although their contribution was notably lower than geographic distance in both sexes (climate: *R* ^2^ = 0.10, *β* = 0.1; body size: *R* ^2^ = 0.07, *β* = 0.33), (Table 1a). The univariate MMRR analysis of plumage color was not statistically significant in the phylogroup or individual‐level analyses (Table 1b). Finally, the univariate analysis of body size was not significant for most of the variables (Table 1c), except for geography, though its contribution was very low in both sexes (males: *R* ^2^ = 0.05, *β* = 0.17; females: *R* ^2^ = 0.02, *β* = 0.12).

**Table 1:** Results of univariate MMRR analysis grouping by sex for analysis between phylogroups and individuals of *Habia rubica*, testing three independent variables of distance: genetics, color, and body size. Here, we show the results of coefficient of determination (*R* ^2^), beta weights (*β*) and *p*‐value (*p*) for each predictor. Because the genetic distances between phylogroups were the same, a single centroid was calculated per phylogroup and the same polygons were obtained for each phylogroup, and the first two results for analysis between phylogroups show the relationship between genetics, geography, and climate of both sexes

	Analysis by individuals						Analysis by phylogroups					
	Males			Females			Males			Females		
	*R* ^2^	*β*	*p*	*R* ^2^	*β*	*p*	*R* ^2^	*β*	*p*	*R* ^2^	*β*	*p*
(a) MRM (mtDNA ~ Predictor)												
Geography	**0.73**	**0.83**	**<.01**	**0.74**	**0.83**	**<.01**	**0.64**	**0.59**	**<.01**			
Climate	**0.09**	**0.13**	**<.01**	**0.10**	**0.16**	**<.01**	0.01	0.05	.62			
Hue total	0.00	0.02	.62	0.00	−0.03	.38	0.03	0.16	.52	0.00	0.02	.92
Hue dorsal	0.00	0.09	<.01	0.00	0.06	.06	0.08	0.27	.21	0.07	0.22	.37
Hue ventral	4E−07	−0.001	.97	0.00	−0.01	.76	0.05	−0.18	.50	0.08	0.26	.29
Chroma total	0.00	−0.04	.18	0.00	−0.06	.08	0.06	0.22	.30	0.01	0.097	.74
Chroma dorsal	0.00	−0.04	.20	0.00	−0.03	.31	0.05	0.20	.33	0.07	0.25	.32
Chroma ventral	0.00	−0.05	.10	0.00	−0.07	.04	0.05	0.20	.48	0.00	−0.03	.95
Volume total	0.00	−0.02	.54	0.00	−0.004	.94	0.01	−0.09	.72	0.02	−0.12	.40
Volume dorsal	0.00	−0.03	.39	0.01	0.068	.05	0.01	−0.10	.78	0.04	−0.15	.56
Volume ventral	3E−08	0.00	1.00	0.00	0.033	.27	0.05	−0.17	.45	0.01	−0.08	.73
Body size	**0.07**	**0.33**	**<.01**	**0.02**	**0.19**	**<.01**	0.18	0.32	.08	0.15	0.32	.09
(b) MRM (Hue ~ Predictor)												
Geography	0.00	0.02	.36	4E−07	−0.00	.99	0.11	0.26	.12	0.04	0.17	.44
Climate	1E−05	0.00	.95	0.01	−0.04	.10	3.88	0.00	.99	0.03	−0.08	.57
mtDNA	0.00	0.01	.63	0.00	−0.02	.42	0.03	0.18	.54	0.00	0.03	.91
Body size	0.01	0.10	.05	0.00	0.05	.39	0.02	0.10	.70	0.00	−0.01	.97
(c) MRM (Chroma ~ Predictor)												
Geography	0.00	−0.04	.26	0.00	−0.04	.30	0.13	0.30	.06	0.04	0.15	.45
Climate	0.00	−0.01	.78	4E−05	−0.00	.92	0.00	0.00	.94	0.00	0.01	.92
mtDNA	0.00	−0.04	.20	0.00	−0.05	.08	0.06	0.28	.31	0.01	0.11	.71
Body size	0.00	−0.03	.63	0.00	0.04	.51	0.00	0.02	.90	0.06	−0.22	.32
(d) MRM (Volume ~ Predictor)												
Geography	0.00	−0.02	.59	1E−05	−0.00	.91	0.00	−0.03	.88	0.00	0.06	.83
Climate	0.00	−0.00	.82	0.00	−0.03	.33	0.04	−0.10	.53	0.02	0.06	.52
mtDNA	0.00	−0.02	.55	1E−05	−0.00	.92	0.01	−0.14	.73	0.02	−0.18	.37
Body size	0.00	0.05	.51	0.01	0.16	.06	0.07	−0.23	.35	0.02	−0.13	.68
(e) MRM (Body size ~ Predictor)												
Geography	0.05	0.17	<.01	0.02	0.12	<.01	0.34	0.56	.05	0.29	0.48	.06
Climate	0.02	0.05	.02	0.00	0.02	.24	0.00	0.02	.93	0.00	0.02	.89
mtDNA	0.07	0.21	<.01	0.02	0.12	<.01	0.18	0.56	.09	0.15	0.48	.09
Hue total	0.01	0.09	.05	0.00	0.04	.41	0.02	0.16	.71	0.00	−0.01	.97
Hue dorsal	0.00	1.05	.16	0.00	0.01	.77	0.01	0.12	.77	0.00	0.04	.92
Hue ventral	0.00	0.02	.74	4E−05	−0.01	.89	0.01	−0.10	.75	0.13	0.39	.34
Chroma total	0.00	−0.02	.64	0.00	0.03	.52	0.00	0.03	.89	0.06	−0.27	.33
Chroma dorsal	0.00	−0.01	.77	0.00	0.05	.25	0.00	−0.05	.85	0.00	0.02	.95
Chroma ventral	0.00	−0.04	.37	0.00	0.05	.33	0.02	0.16	.70	0.12	−0.39	.21
Volume total	0.00	0.03	.53	0.01	0.08	.08	0.07	−0.27	.36	0.02	−0.13	.67
Volume dorsal	0.00	−0.02	.66	0.00	−0.01	.86	0.06	−0.27	.42	0.04	−0.17	.55
Volume ventral	0.00	−0.01	.82	0.00	−0.01	.78	0.07	−0.26	.35	0.02	−0.14	.61

In bold, high beta values were highlighted that were significant in the test.

### 3.4 MMR method for multivariate analysis

**Figure 4:**
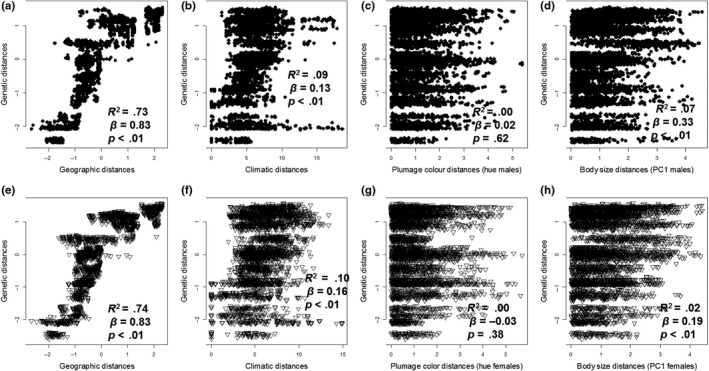
Pairwise distance matrices of mitochondrial DNA (mtDNA) against geographic distance, climate, color, and body size between individuals of *H. rubica* (black points (a–d): phenotypic data of males; hollow triangles (e–h): phenotypic data of females). Beta weights (*β*) and *p*‐value (*p*) of each relationship tested are shown on the graph

In general, the multivariate model explained a high percentage of the total variance in genetic distance at both the phylogroup (*R* ^2^ = 0.66) and individual levels (*R* ^2^ = 0.74, Figure 4). By far, the single most important predictor of genetic distance was geographic distance, and in the phylogroup‐level analysis, it was the only significant predictor variable. Geographic distance accounted for between 63% and 81% of the total variance explained by the multivariate models (Table 2a, Appendix S3: Table S3.6), while climate, plumage color, and body size variables each accounted for less than 10% of the variance explained by the overall model. In the individual‐level analyses, climate and body size had significant effects on genetic variation in males and only climate variables affected genetic variation in female, but the effects were weak overall. Our results did not differ when different plumage color metrics were used (Table 2b).

**Table 2:** Results of multivariate MMRR analysis grouped by sex for analysis between phylogroups and individuals of *Habia rubica*. We show the results of coefficient of determination (*R* ^2^) Beta weights (*β*) and *p*‐value (*p*) and of each predictor from the overall model

	Analysis by individuals						Analysis by phylogroups					
	Males			Females			Males			Females		
	*R* ^2^	*β*	*p*	*R* ^2^	*Β*	*p*	*R* ^2^	*β*	*p*	*R* ^2^	*β*	*p*
(a) MRM (mtDNA ~ Geography + Clime + Hue + Body size)												
Geography	0.74	0.80	<.01	0.74	0.81	<.01	0.66	0.64	<0.01	0.66	0.64	<.01
Climate		0.03	<.01		0.03	<.01		0.01	0.86		0.00	.99
Hue		−0.02	.17		−0.02	.19		−0.11	0.56		−0.13	.46
Body size		0.09	<.01		0.02	.14		−0.06	0.64		−0.06	.67
(b) MRM (Hue ~ Geography + Clime + mtDNA + Body size)												
Geography	0.01	0.06	.35	0.012	0.07	.24	0.15	0.50	0.17	0.15	0.54	.17
Climate		−0.003	.88		−0.04	.12		−0.01	0.93		−0.09	.56
mtDNA		−0.06	.27		−0.06	.19		−0.30	0.52		−0.44	.38
Body size		0.10	.06		0.05	.37		−0.10	0.70		−0.18	.52
(c) MRM (Chroma ~ Geography + Clime + mtDNA + Body size)												
Geography	0.00	−0.03	.70	0.00	0.02	.82	0.20	0.54	0.13	0.23	0.50	.14
Climate		−0.00	.97		0.00	.86		−0.01	0.91		0.00	.97
mtDNA		−0.00	.93		−0.07	.22		−0.17	0.70		−0.22	.57
Body size		−0.02	.75		0.05	.42		−0.24	0.33		−0.45	.07
(d) MRM (Volume ~ Geography + Clime + mtDNA + Body size)												
Geography	0.00	−0.01	.85	0.01	−0.01	.90	0.15	0.36	0.34	0.20	0.62	.11
Climate		−0.00	.86		−0.04	.33		−0.09	0.58		0.07	.51
mtDNA		−0.02	.73		0.00	.93		−0.31	0.52		−0.75	.09
Body size		0.07	.45		0.17	.10		−0.35	0.26		−0.27	.27
(e) MRM (Body size ~ Geography + Clime + mtDNA + Hue)												
Geography	0.08	0.01	.88	0.027	0.07	.16	0.35	0.71	0.16	0.31	0.62	.19
Climate		0.02	.24		0.00	.86		−0.01	0.95		−0.02	.90
mtDNA		0.18	<.01		0.06	.17		−0.19	0.70		−0.19	.72
Hue		0.09	.05		0.04	.35		−0.11	0.74		−0.16	.63

In the comparison between the univariate and multivariate results obtained through the data matching (Tables 1 and 2) and through the matrices obtained for Mexico (Appendix: Table S3.3 and S3.4), no significant differences were observed, given that in this last analysis also was the geographic distance the factor that explains the greatest proportion of the genetic variation found in *H. rubica* (*R* ^2^ = 0.31, *β* = 0.50 for males and *R* ^2^ = 0.48, *β* = 0.86 for females). However, the environmental difference also proved to be an important factor (*R* ^2^ = 0.34, *β* = 0.18 for males and *R* ^2^ = 0.23, *β* = 0.22 for females). In the case of the regressions made with data grouped by phylogroups, the contribution of the factors was not significant.

## 4 DISCUSSION

Our results provide strong evidence that geographic distance is a major driver of genetic variation in *H. rubica*. We did not find evidence that climate variation or phenotypic variation (i.e., body size and plumage coloration) is driving population differentiation in this species complex over its large geographic distribution.

### 4.1 Isolation by distance

Multiple Matrix Regression with Randomization analyses revealed that geographic distance was the predominant factor explaining patterns of deep genetic differentiation across populations of *H. rubica* (Ramírez‐Barrera et al., 2018). This result is consistent between the phylogroup‐level and individual‐level analyses (Tables 1 and 2). These results suggest that population differentiation in *H. rubica* might be explained mostly by a process of isolation by distance (IBD, Wright, 1943). Under this process, the observed genetic structure suggests equilibrium between gene flow and drift (Hutchison & Templeton, 1999) that could be explained by two processes: long‐distance movement and local dispersal (Malpica & Ornelas, 2013).

It is generally accepted that IBD is one of the main factors driving genetic divergence in natural populations (Wu, Yu, & Xu, 2016). Since IBD considers the role of geographic barriers in the process of genetic differentiation among populations in addition to distance per se, patterns of differentiation can provide information on the historical patterns of dispersal by the taxon (Garrido‐Garduño & Vázquez‐Domínguez, 2013; Slatkin, 1994). Species diversification can therefore be strongly influenced by processes such as plate tectonics and climate change that promote speciation by vicariance, as well as speciation by dispersal events. The effects of the paleogeographic changes in the Miocene and Pliocene on speciation trends in neotropical birds are related to the formation and disappearance of barriers and bridges, which influence and even change migration and isolation patterns that favor vicariance (Coyne & Orr, 2004; Rull, 2008). The complicated phylogeographic structure of *H. rubica* is consistent with some geological and biogeographic characteristics of their distribution that could limit gene flow between remote populations (Lavinia et al., 2015; Ramírez‐Barrera et al., 2018).

The phylogeographic structure of the *H. rubica* species complex can be grouped in seven phylogroups (Ramírez‐Barrera et al., 2018), five of which are distributed in Mesoamerica (i.e., the region between Central Mexico and Western Panama; García‐Moreno, Cortés, García‐Deras, & Hernández‐Baños, 2006) and two of which are in South America (Figure 2a,g). Given the large number of phylogroups in a comparatively small area, Mesoamerica can be considered a hot spot for this species, where differentiation among populations has occurred in a relatively short time period. Molecular evidence has shown a similar pattern in the plants of Central America, which originated more recently than South America taxa (Pennington et al., 2004; Pennington, Prado, & Pendry, 2000). The five Mesoamerican phylogroups of *H. rubica* are distributed in the northern (NP) and southern (SP) regions of the Mexican Pacific coast, on the slope of the Gulf of Mexico (GM), from Southeastern Mexico to Costa Rica (SE), and Panama (PA). Mesoamerica has been described as a highly fragmented topographic complex where the composition of flora and fauna has been strongly influenced by both climatic and geological events (Burnham & Graham, 1999; Coates & Obando, 1996). These events have given rise to geographic characteristics such as the Balsas River and the Isthmus of Tehuantepec in Mexico and the Central American Volcanic Arc and the Isthmus of Panama in Central America, which could drive the high ecological diversity of this region.

The other two phylogroups of *H. rubica* are distributed in Western (WS) and Eastern/Northwestern (ES) South America (Lavinia et al., 2015; Ramírez‐Barrera et al., 2018). We suggest that the association between the populations from Atlantic forests and northwestern South America (phylogroup ES) could indicate that the evolutionary history of these populations is deeply associated with those reported for the seasonal forests of South America (Banda et al., 2016; Lavinia et al., 2015; Pennington, Lavin, & Oliveira‐Filho, 2009; Pennington et al., 2004, 2000; Prates et al., 2017). The rainforests of the Amazon basin and the tropical forests of the Atlantic are two of the most important morphoclimatic domains of South America (Ab'Saber, 1977). These two forests are separated by a diagonal strip of dry vegetation, a corridor considered an important barrier for the migration of species between the two forest regions (Por, 1992). However, vegetation maps show that gallery forests and forests distributed in patches across the dry diagonal constitute an interconnected network (Oliveira‐Filho & Ratter, 1995). In addition, several lines of evidence support the hypothesis of old contact between the two regions through this strip of dry vegetation (Auler et al., 2004; Costa, 2003; Oliveira, Barreto, & Suguio, 1999; Por, 1992; Wang et al., 2004). To explain this contact between the eastern and western regions of South America, at least two main routes have been suggested. The first, which arose during the middle to late Miocene, extended through the current Cerrado and Mato Grosso regions of Brazil (Hulka, Grafe, Sames, Uba, & Heubeck, 2006; Roddaz et al., 2006); the second, during the Pliocene and Pleistocene, extended through the current Cerrado and Caatinga regions of northeastern Brazil (Auler et al., 2004; Costa, 2003; Por, 1992; Wang et al., 2004), as a result of the expansion of the gallery forests during the Quaternary climate changes. Some studies have suggested the existence of these old connections in lizard species (Pellegrino, Rodriguez, Harris, Yonenaga‐Yassuda, & Sites, 2011; Prates et al., 2017), mammals (Galewski, Mauffrey, Leite, Patton, & Douzery, 2005), and birds (Lavinia et al., 2015). In support of the latter, there is evidence of genetic divergence during the Pleistocene, following the route of expansion of dry habitats between the two biomes (Martins, Templeton, Pavan, Kohlbach, & Morgante, 2009). This hypothesis of the evolution of the vegetation in South American could explain the pattern in *H. rubica* where, as mentioned before, two phylogroups are defined in this area and coincide with the separation of the Amazonian forest from the Atlantic forest as well as the connection between the Atlantic forest and the northwest populations (Banda et al., 2016; Pennington et al., 2009, 2004, 2000; Prates et al., 2017; Ramírez‐Barrera et al., 2018). All of the geographic features mentioned above are considered important barriers to dispersal in several animal taxa (Amman & Bradley, 2004; Bryson, García‐Vázquez, & Riddle, 2011; BrysonJr, Nieto‐Montes de Oca, & Reyes, 2008; Daza, Castoe, & Parkinson, 2010; Devitt, 2006; Gutiérrez‐García & Vázquez‐Domínguez, 2012, 2013; Navarro‐Sigüenza, Peterson, Nyari, García‐Deras, & García‐Moreno, 2008; Suárez‐Atilano, Burbrink, & Vázquez‐Domínguez, 2014).

### 4.2 Isolation by environment

Environmental dissimilarity did not have a significant effect on genetic differentiation of *H. rubica* after controlling for geographic distance (Table 2). This suggests that geographic isolation (i.e., isolation by distance, IBD; Wright, 1943) but not adaptation to local climatic environments (i.e., isolation by environmental, IBE; Wang & Bradburd, 2014) was the underlying process of the observed patterns of genetic structure (Figure 2g).

This suggests that climate likely is not playing a major role in genetic differentiation within *H. rubica*. However, climatic fluctuations seem to have played a major role in the diversification history of the species. This is also evidenced by the fact that there is a single phylogroup formed in the Gulf of Mexico region, despite a vast range of environmental conditions, from dry forests in Tamaulipas to Rainforest in Veracruz. This might be explained if we consider that even through a very broad distribution area. Therefore, there was little association between genetic differentiation and climatic differentiation. This phylogeographic pattern of *H. rubica* has been previously reported in several species from regions with more contrasting climatic fluctuations in South America (Carnaval, Hickerson, Haddad, Rodrigues, & Moritz, 2009; Yannik et al., 2014).

On the other hand, the lack of signal in isolation by environment analyses in *H. rubica* could occur for other reasons, including adaptation to local environments through phenotypic plasticity (Ramírez‐Valiente, Sanchez‐Gomez, Aranda, & Valladares, 2010), positive selection on immigrant genotypes from distant populations mediated by heterosis (Bensch et al., 2006), or as a consequence of long‐distance gene flow counteracting the effects of natural selection and impeding or attenuating local adaptation processes (Buschbom, Yanbaev, & Degen, 2011). It should be noted that we cannot rule out all hypothesis of isolation by adaptation and isolation by environment, given that other parameters (e.g., vocal variation, other attributes of coloration, vegetation) were not considered in our study and could potentially affect genetic differentiation within the *H. rubica* complex (Lavinia et al., 2015; Ramírez‐Barrera et al., 2018).

### 4.3 Local adaptation

While our results show a positive correlation between biogeographic patterns of diversification and phenotypic divergence (plumage coloration and body size), the MMRR analysis does not provide enough evidence to support ecological speciation.

Even though plumage differentiation is often considered a relevant character for species delimitation in avian taxonomy, in some cases it does not provide enough evidence for the correct discrimination of species. *Habia rubica* has considerable plumage color differentiation, but this appears to be a result of neutral processes (e.g., genetic drift). We found little support for the role of plumage divergence in explaining genetic divergence (i.e., isolation by adaptation).

Body size clines that are correlated with temperature gradients are common in nature, particularly in birds (Friedman & Remeš, 2016; Meiri & Dayan, 2003). These correlations are often taken as evidence of local thermoregulatory adaptation (Friedman & Remeš, 2017). However, the precise selective agent is debatable, as many variables that plausibly correlate with variation in body mass also covary with elevation, temperature gradients, or latitude (Seeholzer & Brumfield, 2017). Although body size divergence is likely influenced by environmental divergence, it has not impacted genetic structure in *H. rubica* and, in practice, body size is generally not ultimately an important character in avian species delimitation (Price, 2007). However, phenotypic variation at the intraspecific level may present high correlation with genetic variation (García, Barreira, Lavinia, & Tubaro, 2016).

Plumage and vocal differences are expected to play a more important role in conspecific recognition and mate choice in birds than body size (Hilty, 2011; Lavinia et al., 2015; Price, 2007) and thus may be important in structuring genetic variation (Seeholzer & Brumfield, 2017). It is expected that rapid local adaptation and phenotypic divergence will occur at the edges of range expansions (García‐Ramos & Kirkpatrik, 1997), which Mayr proposed as an important driver of incipient speciation (Mayr, 1982).

## CONFLICT OF INTEREST

None declared.

## AUTHOR CONTRIBUTIONS

SM Ramírez‐Barrera conceived the ideas, designed and performed the research, analyzed data, prepared figures and/or tables and reviewed drafts of the paper. JA Velasco analyzed, interpreted data and reviewed drafts of the paper. T Orozco‐Tellez collected part of the data. AM Vázquez‐López collected the data. BE Hernández‐Baños conceived the ideas, designed the research, contributed reagents/materials/analysis tools and reviewed drafts of the paper.

## Data Availability

Sampling locations, morphological, and coloration data: We have curation plans before archiving the data. These curation plans include editing and organizing the morphology and coloration data. The data were grouped by filogroup (geographic region) and is now available for consultation at: *DNA sequences*: https://figshare.com/s/fe9f9f6fed1686782f62; *Morphological data*: https://doi.org/10.6084/m9.figshare.8023565; *Coloration data by phylogroups*: *NP*, northern pacific of Mexico (https://doi.org/10.6084/m9.figshare.9883382); *SP*, southern pacific of Mexico (https://doi.org/10.6084/m9.figshare.9883388); *GM*, Gulf of Mexico (https://doi.org/10.6084/m9.figshare.9883391); *SE*, southeastern Mexico and northern Central America (https://doi.org/10.6084/m9.figshare.9883430; https://doi.org/10.6084/m9.figshare.9883511); *PA*, Panama (https://doi.org/10.6084/m9.figshare.9883514); *WS*, western South America (https://doi.org/10.6084/m9.figshare.9883556; https://doi.org/10.6084/m9.figshare.9883562); *ES*, eastern‐northwestern South America (https://doi.org/10.6084/m9.figshare.9883523).
