# Supplementary material for: What drives genetic and phenotypic divergence in the Red‐crowned Ant tanager (Habia rubica, Aves: Cardinalidae), a polytypic species?
Source: Ecol Evol. 2019 Oct 21;9(21):12339–52. doi: 10.1002/ece3.5742 (PMC6854386; doi:10.1002/ece3.5742)
Supplement: Supplementary file 1 [file ECE3-9-12339-s001.docx]

*Ecology & Evolution*

**SUPPLEMENTARY MATERIAL**

**What drives genetic and phenotypic divergence in the Red-crowned Ant-tanager (*Habia rubica*, Aves: Cardinalidae), a polytypic species?**

Sandra M. Ramírez-Barrera ^1,3^, Julián A. Velasco ^2^, Tania^2^ Orozco-Téllez, Alma M. Vázquez-López ^1,3^ & Blanca E. Hernández-Baños ^3*^

^1^Posgrado en Ciencias Biológicas, Universidad Nacional Autónoma de México, México, CMX., México

^2^Centro de Ciencias de la Atmósfera, Universidad Nacional Autónoma de México, Ciudad de México, México

^3^Departamento de Biología Evolutiva, Facultad de Ciencias, Museo de Zoología, Universidad Nacional Autónoma de México, Ciudad de México, México

To complement the information given in the main manuscript, the following sections provide Supplementary information for material (Appendix S1), and results (Appendix S2).

**Appendix S1**

Supporting information on the material used in the study

**Table S1.1.** List of total samples used in this study and pairing of genetic and phenotypic data (column genetic association). The collections of origin of each sample are: Museo de Zoología “Alfonso L. Herrera”, UNAM, Mexico (MZFC); Colección Nacional de Aves, UNAM, Mexico (CNAV); El Colegio de la Frontera Sur, Chetumal, México (ECOSUR-Ch); the Ornithological Collection of the American Museum of Natural History, New York (AMNH), the Ornithological Collection of the Smithsonian Institution, Washington D. C (SI); The Burke Museum, University of Washington (UWBM); Natural History Museum of The University of Kansas (KU) and Museum of Natural Science of Louisiana State University (LSU). The abbreviation SEC responds with sequences of the mitochondrial gene ND2 provided by John Klicka. The phylogroup column refers to the names of each genetic groups identified in *Habia rubica*: NP, northern pacific of Mexico; SP, southern pacific of Mexico; GM, Gulf of Mexico; SE, southeastern Mexico and northern Central America; PA, Panama; WS, western South America and ES, eastern-northwestern South America.

| Specimen data | | | | | | | Phenotypic data | Genetic asociation | |
| --- | --- | --- | --- | --- | --- | --- | --- | --- | --- |
| Collection number | Sex | Collection | Latitude | Longitude | Country | State |  | Phylogroup | Collection number |
| 156119 | F | SI | 20.76 | -104.85 | Mexico | Jalisco | x | NP | URRA67 |
| 156120 | F | SI | 20.76 | -104.85 | Mexico | Jalisco | x | NP |  |
| 510473 | M | AMNH | 20.81 | -105.25 | Mexico | Nayarit | x | NP | CONA799 |
| 510475 | F | AMNH | 21.83 | -103.78 | Mexico | Jalisco | x | NP |  |
| 510476 | M | AMNH | 21.83 | -103.78 | Mexico | Jalisco | x | NP |  |
| P9771 | F | CNAV | 21.15 | -105.12 | Mexico | Nayarit | x | NP | URRA63 |
| P9772 | M | CNAV | 21.15 | -105.12 | Mexico | Nayarit | x | NP | URRA63 |
| P9773 | F | CNAV | 21.30 | -105.10 | Mexico | Nayarit | x | NP |  |
| P9774 | F | CNAV | 20.22 | -103.70 | Mexico | Jalisco | x | NP |  |
| P22391 | M | CNAV | 22.39 | -105.27 | Mexico | Nayarit | x | NP |  |
| P22392 | M | CNAV | 20.61 | -105.23 | Mexico | Jalisco | x | NP | URRA55 |
| P22393 | M | CNAV | 19.17 | -103.94 | Mexico | Colima | x | NP |  |
| PEP459 | M | MZFC | 20.75 | -105.38 | Mexico | Nayarit | x | NP | URRA60 |
| URRA55 | F | MZFC | 20.47 | -105.29 | Mexico | Jalisco | x | NP | URRA55 |
| URRA60 | F | MZFC | 20.47 | -105.29 | Mexico | Jalisco | x | NP | URRA60 |
| URRA67 | M | MZFC | 20.47 | -105.29 | Mexico | Jalisco | x | NP | URRA67 |
| URRA63 | ND | MZFC | 20.47 | -105.29 | Mexico | Jalisco | x | NP |  |
| CONACYT799 | F | MZFC | 19.46 | -103.71 | Mexico | Colima | x | NP | CONA799 |
| FRG116 | M | MZFC | 21.58 | -105.23 | Mexico | Nayarit | x | NP |  |
| FRG76 | M | MZFC | 21.44 | -105.00 | Mexico | Nayarit | x | NP |  |
| 185673 | F | SI | 17.04 | -100.25 | Mexico | Guerrero | x | SP | MOLGRO454 |
| 185674 | M | SI | 17.04 | -100.25 | Mexico | Guerrero | x | SP | MOLGRO455 |
| P9775 | M | CNAV | 18.75 | -102.37 | Mexico | Michoacán | x | SP | MOLGRO454 |
| P9776 | F | CNAV | 18.75 | -102.37 | Mexico | Michoacán | x | SP | MICH196 |
| P9777 | M | CNAV | 16.17 | -97.10 | Mexico | Oaxaca | x | SP | OMVP161 |
| P9778 | F | CNAV | 16.17 | -97.10 | Mexico | Oaxaca | x | SP |  |
| P9779 | M | CNAV | 16.16 | -97.13 | Mexico | Oaxaca | x | SP | OMVP672 |
| P9780 | M | CNAV | 16.16 | -97.13 | Mexico | Oaxaca | x | SP | OAX58 |
| P9781 | F | CNAV | 16.16 | -97.13 | Mexico | Oaxaca | x | SP |  |
| P13206 | M | CNAV | 16.22 | -97.23 | Mexico | Oaxaca | x | SP | OMVP160 |
| P13207 | F | CNAV | 16.22 | -97.23 | Mexico | Oaxaca | x | SP | OMVP682 |
| P15757 | M | CNAV | 16.13 | -97.10 | Mexico | Oaxaca | x | SP |  |
| P28151 | F | CNAV | 18.28 | -102.58 | Mexico | Michoacán | x | SP | MICH126 |
| PLU17 | M | MZFC | 15.89 | -96.40 | Mexico | Oaxaca | x | SP | MIA81 |
| PLU18 | F | MZFC | 15.89 | -96.40 | Mexico | Oaxaca | x | SP | OMVP160 |
| PLU23 | M | MZFC | 15.89 | -96.40 | Mexico | Oaxaca | x | SP | OAX115 |
| SRSC024 | M | MZFC | 17.23 | -99.86 | Mexico | Guerrero | x | SP | MOLGRO41 |
| AGNS404 | M | MZFC | 17.25 | -100.30 | Mexico | Guerrero | x | SP | MOLGRO43 |
| AGNS405 | F | MZFC | 17.25 | -100.30 | Mexico | Guerrero | x | SP | MOLGRO40 |
| AMT382 | F | MZFC | 15.93 | -96.42 | Mexico | Oaxaca | x | SP | MOLGRO1008 |
| AZAR09 | M | MZFC | 18.19 | -100.16 | Mexico | Guerrero | x | SP | MOLGRO262 |
| MOLGRO40 | M | MZFC | 17.36 | -99.46 | Mexico | Guerrero | x | SP | MOLGRO40 |
| MOLGRO41 | F | MZFC | 17.36 | -99.46 | Mexico | Guerrero | x | SP | MOLGRO41 |
| MOLGRO42 | F | MZFC | 17.36 | -99.46 | Mexico | Guerrero | x | SP | MOLGRO42 |
| MOLGRO43 | F | MZFC | 17.36 | -99.46 | Mexico | Guerrero | x | SP | MOLGRO43 |
| MOLGRO261 | M | MZFC | 17.59 | -99.84 | Mexico | Guerrero | x | SP | MOLGRO261 |
| MOLGRO262 | F | MZFC | 17.59 | -99.84 | Mexico | Guerrero | x | SP | MOLGRO262 |
| MOLGRO438 | F | MZFC | 17.53 | -100.56 | Mexico | Guerrero | x | SP |  |
| MOLGRO440 | F | MZFC | 17.53 | -100.56 | Mexico | Guerrero | x | SP | MOLGRO261 |
| molgro454 | ND | MZFC | 17.53 | -101.44 | Mexico | Guerrero | x | SP |  |
| MOLGRO455 | F | MZFC | 17.18 | -99.49 | Mexico | Guerrero | x | SP | MOLGRO455 |
| MOLGRO591 | M | MZFC | 17.36 | -99.54 | Mexico | Guerrero |  | SP |  |
| omvp160 | ND | MZFC | 16.96 | -97.91 | Mexico | Oaxaca |  | SP |  |
| mia81/amt390 | ND | MZFC | 15.93 | -96.42 | Mexico | Oaxaca |  | SP |  |
| OMVP161 | F | MZFC | 16.96 | -97.91 | Mexico | Oaxaca | x | SP | OMVP161 |
| OMVP672 | F | MZFC | 16.83 | -97.88 | Mexico | Oaxaca | x | SP | OMVP672 |
| OMVP682 | M | MZFC | 16.82 | -97.89 | Mexico | Oaxaca | x | SP | OMVP682 |
| OMVP1008 | M | MZFC | 16.24 | -97.29 | Mexico | Oaxaca | x | SP | MOLGRO1008 |
| oaxjk07115 | ND | UWBM | 16.20 | -97.15 | Mexico | Oaxaca |  | SP |  |
| oaxjk07058 | ND | UWBM | 16.20 | -97.15 | Mexico | Oaxaca |  | SP |  |
| michbts08126 | ND | UWBM | 18.10 | -102.40 | Mexico | Michoacán |  | SP |  |
| michbts08196 | ND | UWBM | 18.17 | -102.31 | Mexico | Michoacán |  | SP |  |
| 143576 | M | SI | 18.64 | -96.73 | Mexico | Veracruz | x | GM |  |
| 158612 | M | SI | 20.73 | -97.85 | Mexico | Puebla | x | GM |  |
| 158614 | F | SI | 20.73 | -97.85 | Mexico | Puebla | x | GM |  |
| 158615 | F | SI | 20.73 | -97.85 | Mexico | Puebla | x | GM |  |
| 370781 | F | SI | 19.20 | -96.16 | Mexico | Veracruz | x | GM |  |
| 95901 | M | AMNH | 18.64 | -96.73 | Mexico | Veracruz | x | GM | NAR28 |
| 153421 | F | AMNH | 19.54 | -96.91 | Mexico | Veracruz | x | GM |  |
| 707207 | M | AMNH | 19.15 | -96.17 | Mexico | Veracruz | x | GM |  |
| P7664 | F | CNAV | 18.45 | -95.22 | Mexico | Veracruz | x | GM |  |
| P7677 | M | CNAV | 18.58 | -95.07 | Mexico | Veracruz | x | GM |  |
| P9782 | F | CNAV | 18.60 | -95.07 | Mexico | Veracruz | x | GM | TUX37 |
| P9783 | M | CNAV | 18.58 | -95.07 | Mexico | Veracruz | x | GM | TXT15 |
| P15699 | M | CNAV | 17.11 | -95.03 | Mexico | Oaxaca | x | GM |  |
| P15711 | F | CNAV | 18.42 | -95.07 | Mexico | Veracruz | x | GM | 28txt15 |
| P15712 | F | CNAV | 18.47 | -95.35 | Mexico | Veracruz |  | GM |  |
| P15730 | F | CNAV | 18.97 | -97.07 | Mexico | Veracruz | x | GM | NAR28 |
| P15735 | F | CNAV | 17.50 | -94.92 | Mexico | Veracruz |  | GM |  |
| P15736 | F | CNAV | 17.17 | -95.03 | Mexico | Oaxaca | x | GM |  |
| P15737 | F | CNAV | 17.17 | -95.03 | Mexico | Oaxaca | x | GM | OMVP574 |
| P15738 | M | CNAV | 17.17 | -95.03 | Mexico | Oaxaca | x | GM |  |
| P15739 | M | CNAV | 17.37 | -95.05 | Mexico | Veracruz | x | GM |  |
| P15741 | M | CNAV | 17.17 | -95.03 | Mexico | Oaxaca | x | GM |  |
| P15742 | F | CNAV | 17.83 | -95.82 | Mexico | Veracruz | x | GM | 29txt19 |
| P15745 | M | CNAV | 17.37 | -95.05 | Mexico | Veracruz | x | GM |  |
| P15760 | M | CNAV | 17.17 | -95.03 | Mexico | Oaxaca | x | GM |  |
| P15761 | M | CNAV | 17.17 | -95.03 | Mexico | Oaxaca | x | GM |  |
| P15762 | M | CNAV | 17.17 | -95.03 | Mexico | Oaxaca | x | GM |  |
| P24436 | F | CNAV | 18.43 | -94.91 | Mexico | Veracruz |  | GM |  |
| P24437 | F | CNAV | 18.42 | -95.12 | Mexico | Veracruz | x | GM |  |
| P24438 | M | CNAV | 18.34 | -94.89 | Mexico | Veracruz | x | GM |  |
| P24441 | M | CNAV | 18.34 | -94.89 | Mexico | Veracruz | x | GM |  |
| P24442 | F | CNAV | 18.25 | -94.86 | Mexico | Veracruz | x | GM |  |
| P24443 | F | CNAV | 18.27 | -95.70 | Mexico | Veracruz | x | GM |  |
| P24445 | M | CNAV | 18.58 | -95.05 | Mexico | Veracruz | x | GM |  |
| P24497 | M | CNAV | 18.58 | -95.05 | Mexico | Veracruz | x | GM |  |
| P24593 | F | CNAV | 18.29 | -93.86 | Mexico | Veracruz | x | GM |  |
| P24594 | M | CNAV | 18.29 | -93.86 | Mexico | Veracruz | x | GM |  |
| P27809 | M | CNAV | 17.17 | -95.03 | Mexico | Oaxaca |  | GM |  |
| P29301 | M | CNAV | 18.43 | -94.91 | Mexico | Veracruz | x | GM | TXT19 |
| P29491 | M | CNAV | 18.43 | -94.91 | Mexico | Veracruz | x | GM | VER343363 |
| P29494 | M | CNAV | 18.43 | -94.91 | Mexico | Veracruz | x | GM | VER393884 |
| P29507 | M | CNAV | 18.43 | 94.91 | Mexico | Veracruz | x | GM |  |
| P29530 | F | CNAV | 18.43 | 94.91 | Mexico | Veracruz | x | GM | VER393884 |
| P29535 | M | CNAV | 18.43 | 94.91 | Mexico | Veracruz | x | GM |  |
| P29542 | M | CNAV | 18.43 | 94.91 | Mexico | Veracruz | x | GM |  |
| P29543 | M | CNAV | 18.43 | 94.91 | Mexico | Veracruz | x | GM |  |
| P29547 | M | CNAV | 18.43 | -94.97 | Mexico | Veracruz | x | GM |  |
| P29548 | M | CNAV | 18.43 | -94.97 | Mexico | Veracruz | x | GM |  |
| 11095 | M | MZFC | 18.58 | -95.07 | Mexico | Veracruz | x | GM |  |
| AGNS1004 | M | MZFC | 17.71 | -96.41 | Mexico | Oaxaca | x | GM |  |
| AGNS1007 | F | MZFC | 17.71 | -96.41 | Mexico | Oaxaca | x | GM |  |
| AGNS1010 | M | MZFC | 17.71 | -96.41 | Mexico | Oaxaca | x | GM |  |
| AMT169 | F | MZFC | 21.07 | -98.99 | Mexico | Hidalgo | x | GM |  |
| AMT241 | M | MZFC | 20.73 | -98.16 | Mexico | Veracruz | x | GM | HGOSLP143 |
| AV328 | M | MZFC | 19.18 | -96.1429000 | Mexico | Veracruz | x | GM |  |
| CHIMA86 | F | MZFC | 17.07 | -94.12 | Mexico | Oaxaca | x | GM |  |
| CHIMA162 | F | MZFC | 17.07 | -94.58 | Mexico | Oaxaca | x | GM | OMVP563 |
| CHIMA164 | M | MZFC | 17.07 | -94.58 | Mexico | Oaxaca | x | GM | OMVP545 |
| CHIMA416 | F | MZFC | 17.07 | -94.05 | Mexico | Oaxaca | x | GM | CHIMA416 |
| CHIMA516 | M | MZFC | 17.01 | -94.69 | Mexico | Oaxaca | x | GM | OMVP546 |
| CHIMA526 | M | MZFC | 17.01 | -94.69 | Mexico | Oaxaca | x | GM |  |
| CHIMAS112 | M | MZFC | 17.07 | -94.12 | Mexico | Oaxaca | x | GM | CHIMA416 |
| HGO-SLP143 | F | MZFC | 21.08 | -98.96 | Mexico | Hidalgo | x | GM | HGOSLP143 |
| MEX035 | F | MZFC | 18.32 | -94.83 | Mexico | Veracruz | x | GM | TXT58 |
| MEX056 | F | MZFC | 18.32 | -94.83 | Mexico | Veracruz | x | GM | VER343363 |
| MEX123 | M | MZFC | 18.32 | -94.83 | Mexico | Veracruz | x | GM | TXT62 |
| MEX133 | F | MZFC | 18.32 | -94.83 | Mexico | Veracruz | x | GM | TXT63 |
| MEX134 | F | MZFC | 18.32 | -94.83 | Mexico | Veracruz | x | GM | TXT64 |
| OMVP545 | F | MZFC | 17.07 | -94.58 | Mexico | Oaxaca | x | GM | OMVP545 |
| OMVP546 | F | MZFC | 17.07 | -94.58 | Mexico | Oaxaca | x | GM | OMVP546 |
| OMVP563 | M | MZFC | 17.02 | -94.66 | Mexico | Oaxaca | x | GM | OMVP563 |
| OMVP574 | M | MZFC | 17.02 | -94.66 | Mexico | Oaxaca | x | GM | OMVP574 |
| NAR 28 | ND | MZFC | 18.80 | -96.96 | Mexico | Veracruz |  | GM |  |
| TUXFPO37 | M | MZFC | 18.59 | -95.10 | Mexico | Veracruz | x | GM | TUXFPO37 |
| TXT58 | M | MZFC | 18.31 | -94.88 | Mexico | Veracruz | x | GM | TXT58 |
| TXT62 | F | MZFC | 18.31 | -94.88 | Mexico | Veracruz | x | GM | TXT62 |
| TXT63 | M | MZFC | 18.31 | -94.88 | Mexico | Veracruz | x | GM | TXT63 |
| TXT64 | M | MZFC | 18.31 | -94.88 | Mexico | Veracruz | x | GM | TXT64 |
| TXT15 | ND | MZFC | 18.55 | -95.12 | Mexico | Veracruz |  | GM |  |
| TXT19 | ND | MZFC | 18.55 | -95.12 | Mexico | Veracruz |  | GM |  |
| Ver343363 | ND | UWBM | 18.32 | -94.83 | Mexico | Veracruz |  | GM |  |
| Ver393884 | ND | UWBM | 18.32 | -94.83 | Mexico | Veracruz |  | GM |  |
| 35249 | M | SI | 10.07 | -84.31 | Costa Rica |  | x | SE |  |
| 35256 | F | SI | 10.07 | -84.31 | Costa Rica |  | x | SE |  |
| 55935 | M | SI | 9.93 | -84.09 | Costa Rica |  | x | SE |  |
| 161650 | M | SI | 15.51 | -87.99 | Honduras |  | x | SE | 434137 |
| 166580 | M | SI | 17.51 | -91.99 | Mexico | Chiapas | x | SE |  |
| 199431 | M | SI | 9.79 | -84.13 | Costa Rica |  | x | SE |  |
| 199963 | M | SI | 9.73 | -85.02 | Costa Rica |  | x | SE |  |
| 199964 | M | SI | 9.80 | -84.23 | Costa Rica |  | x | SE |  |
| 302815 | M | SI | 16.99 | -89.69 | Guatemala |  | x | SE |  |
| 302816 | F | SI | 16.91 | -90.30 | Guatemala |  | x | SE | B2037 |
| 302818 | M | SI | 17.27 | -90.19 | Guatemala |  | x | SE | B2037 |
| 302819 | M | SI | 16.91 | -90.30 | Guatemala |  | x | SE |  |
| 40893 | M | AMNH | 15.78 | -90.23 | Guatemala |  | x | SE | DHB4362 |
| 95318 | M | AMNH | 10.67 | -85.01 | Costa Rica |  | x | SE | ZMUC130351 |
| 95319 | F | AMNH | 10.67 | -85.01 | Costa Rica |  | x | SE | ZMUC130351 |
| 101458 | M | AMNH | 13.21 | -86.11 | Nicaragua | Jinotega | x | SE | DAB1349 |
| 101459 | M | AMNH | 13.21 | -86.11 | Nicaragua | Jinotega | x | SE |  |
| 101460 | F | AMNH | 13.21 | -86.11 | Nicaragua | Jinotega | x | SE | DAB1484 |
| 101461 | F | AMNH | 13.21 | -86.11 | Nicaragua | Jinotega | x | SE | DAB1508 |
| 101462 | M | AMNH | 12.93 | -85.92 | Nicaragua | Matagalpa | x | SE | DAB1486 |
| 101463 | F | AMNH | 12.93 | -85.92 | Nicaragua | Matagalpa | x | SE | DAB1486 |
| 102359 | M | AMNH | 9.00 | -83.33 | Costa Rica |  | x | SE |  |
| 102361 | F | AMNH | 9.00 | -83.33 | Costa Rica |  |  | SE |  |
| 144707 | M | AMNH | 12.93 | -85.92 | Nicaragua | Matagalpa | x | SE | DAB1508 |
| 144708 | M | AMNH | 11.83 | -85.98 | Nicaragua |  | x | SE | DAB1485 |
| 144710 | F | AMNH | 11.83 | -85.98 | Nicaragua |  |  | SE |  |
| 254746 | M | AMNH | 20.36 | -87.34 | Mexico | Quintara Roo | x | SE |  |
| 254747 | F | AMNH | 20.36 | -87.34 | Mexico | Quintara Roo | x | SE | MOL1121 |
| 326507 | M | AMNH | 15.51 | -87.99 | Honduras | Cortes | x | SE | 5985 |
| 326508 | F | AMNH | 15.51 | -87.99 | Honduras | Cortes | x | SE | DAB1349 |
| 328527 | M | AMNH | 15.51 | -87.99 | Honduras | Cortes | x | SE | 5982 |
| 328529 | M | AMNH | 15.51 | -87.99 | Honduras | Cortes | x | SE | 5978 |
| 328530 | F | AMNH | 15.51 | -87.99 | Honduras | Cortes | x | SE | 5974 |
| 328533 | M | AMNH | 15.42 | -88.16 | Honduras | Cortes | x | SE | 5974 |
| 328535 | F | AMNH | 15.49 | -87.93 | Honduras | Cortes | x | SE | GAV2110 |
| 328538 | M | AMNH | 15.49 | -87.93 | Honduras | Cortes | x | SE | GAV2110 |
| 328539 | M | AMNH | 15.49 | -87.93 | Honduras | Cortes | x | SE | GMS145 |
| 328540 | M | AMNH | 15.49 | -87.93 | Honduras | Cortes | x | SE | 434136 |
| 328541 | F | AMNH | 15.49 | -87.93 | Honduras | Cortes | x | SE | GMS145 |
| 328542 | F | AMNH | 15.49 | -87.93 | Honduras |  | x | SE | 434136 |
| 328544 | M | AMNH | 14.07 | -87.19 | Honduras |  | x | SE | 5934 |
| 328545 | M | AMNH | 14.07 | -87.19 | Honduras |  | x | SE | 5937 |
| 328546 | F | AMNH | 14.07 | -87.19 | Honduras |  | x | SE | 5934 |
| 328549 | F | AMNH | 14.07 | -87.19 | Honduras |  | x | SE | 5937 |
| 328550 | M | AMNH | 14.07 | -87.19 | Honduras |  | x | SE | 5949 |
| 328551 | F | AMNH | 14.07 | -87.19 | Honduras |  | x | SE | 5949 |
| 392420 | F | AMNH | 10.75 | -85.15 | Costa Rica |  | x | SE | 5985 |
| 392422 | M | AMNH | 10.75 | -85.15 | Costa Rica |  | x | SE |  |
| 392424 | M | AMNH | 10.75 | -85.15 | Costa Rica |  | x | SE |  |
| 392425 | F | AMNH | 10.75 | -85.15 | Costa Rica |  | x | SE | 434137 |
| 392426 | F | AMNH | 10.75 | -85.15 | Costa Rica |  | x | SE | DAB1485 |
| 398444 | M | AMNH | 15.53 | -89.77 | Guatemala |  | x | SE | DHB4377 |
| 398447 | M | AMNH | 15.53 | -89.77 | Guatemala |  | x | SE | DHB4399 |
| 398448 | F | AMNH | 15.53 | -89.77 | Guatemala |  | x | SE | MOL1122 |
| 398452 | F | AMNH | 15.53 | -89.77 | Guatemala |  | x | SE | DHB4377 |
| 398454 | F | AMNH | 15.53 | -89.77 | Guatemala |  | x | SE | DHB4369 |
| 423582 | M | AMNH | 12.93 | -85.92 | Nicaragua | Matagalpa | x | SE | DAB1484 |
| 510449 | M | AMNH | 9.93 | -84.18 | Costa Rica | San José | x | SE |  |
| 510450 | F | AMNH | 9.00 | -83.33 | Costa Rica |  |  | SE |  |
| 510451 | M | AMNH | 10.66 | -84.26 | Costa Rica |  | x | SE |  |
| 510463 | M | AMNH | 15.59 | -90.15 | Guatemala | Alta Verapaz | x | SE |  |
| 510471 | M | AMNH | 10.75 | -85.15 | Costa Rica |  | x | SE |  |
| 813870 | M | AMNH | 14.30 | -90.79 | Guatemala | Escuintla | x | SE | DHB4369 |
| 813871 | F | AMNH | 14.30 | -90.79 | Guatemala | Escuintla | x | SE | DHB4362 |
| 813872 | F | AMNH | 14.20 | -90.43 | Guatemala |  |  | SE |  |
| P15740 | M | CNAV | 16.95 | -93.45 | Mexico | Chiapas | x | SE |  |
| P15743 | M | CNAV | 16.95 | -93.45 | Mexico | Chiapas | x | SE |  |
| P15756 | M | CNAV | 16.95 | -93.45 | Mexico | Chiapas | x | SE |  |
| P15763 | M | CNAV | 16.90 | -93.30 | Mexico | Chiapas | x | SE |  |
| P15766 | M | CNAV | 16.95 | -93.45 | Mexico | Chiapas | x | SE |  |
| P22395 | F | CNAV | 16.48 | -92.23 | Mexico | Chiapas | x | SE | 5978 |
| P22397 | M | CNAV | 15.51 | -92.89 | Mexico | Chiapas | x | SE |  |
| P25054 | M | CNAV | 17.99 | -92.93 | Mexico | Tabasco | x | SE |  |
| P29570 | F | CNAV | 15.93 | -90.66 | Guatemala | Alta Verapaz | x | SE |  |
| P29586 | M | CNAV | 15.93 | -90.66 | Guatemala | Alta Verapaz | x | SE |  |
| P29599 | M | CNAV | 15.64 | -88.83 | Guatemala |  | x | SE |  |
| P29600 | F | CNAV | 15.64 | -88.83 | Guatemala |  | x | SE |  |
| P29614 | M | CNAV | 15.64 | -88.83 | Guatemala |  | x | SE |  |
| P29615 | M | CNAV | 15.64 | -88.83 | Guatemala |  | x | SE |  |
| P29616 | F | CNAV | 15.64 | -88.83 | Guatemala |  | x | SE |  |
| P29617 | M | CNAV | 15.64 | -88.83 | Guatemala |  | x | SE |  |
| A-0075 | M | ECOSUR-CH | 18.53 | -88.30 | Mexico | Quintara Roo | x | SE | ADAB95289 |
| ADAB 55 | M | ECOSUR-CH | 18.53 | -88.30 | Mexico | Quintara Roo | x | SE |  |
| ADAB 163 | F | ECOSUR-CH | 18.53 | -88.30 | Mexico | Quintara Roo | x | SE | Y408176 |
| ADAB 164 | F | ECOSUR-CH | 18.53 | -88.30 | Mexico | Quintara Roo | x | SE | B2000 |
| ADAB 95099 | F | ECOSUR-CH | 19.39 | -88.08 | Mexico | Quintara Roo | x | SE | ADAB95289 |
| ADAB 95289 | ND | ECOSUR-CH | 19.16 | -87.89 | Mexico | Quintana Roo |  | SE |  |
| ADAB 96465 | M | ECOSUR-CH | 18.53 | -88.30 | Mexico | Quintara Roo | x | SE |  |
| EMFE 80 | M | ECOSUR-CH | 18.53 | -88.30 | Mexico | Quintara Roo | x | SE |  |
| EMFE 389 | M | ECOSUR-CH | 18.53 | -88.30 | Mexico | Quintara Roo | x | SE |  |
| EMFE 403 | M | ECOSUR-CH | 18.53 | -88.30 | Mexico | Quintara Roo | x | SE |  |
| b2000 | M | MZF | 18.59 | -90.26 | Mexico | Campeche | x | SE |  |
| b2037 | ND | MZF | 18.59 | -90.26 | Mexico | Campeche |  | SE |  |
| Y408 176 | M | MZF | 18.02 | -90.32 | Mexico | Campeche | x | SE | Y408176 |
| HBD053 | M | MZF | 17.97 | -88.89 | Mexico | Quintana Roo | x | SE |  |
| MOL1121 | M | MZF | 18.60 | -89.28 | Mexico | Campeche | x | SE | MOL1121 |
| MOL1122 | M | MZF | 18.59 | -89.26 | Mexico | Campeche | x | SE | MOL1122 |
| MOL1149 | M | MZF | 18.60 | -89.28 | Mexico | Quintana Roo | x | SE | MOL1149 |
| PGD038 | F | MZF | 15.48 | -93.04 | Mexico | Chiapas | x | SE | 5982 |
| QROO017 | M | MZF | 21.21 | -87.19 | Mexico | Quintana Roo |  | SE |  |
| YACH330 | M | MZF | 16.91 | -90.98 | Mexico | Chiapas | x | SE | DAB1485 |
| YACH378 | M | MZF | 16.08 | -90.98 | Mexico | Chiapas | x | SE | YACH378 |
| YACH528 | F | MZF | 16.91 | -90.98 | Mexico | Chiapas | x | SE | YACH528 |
| 13725 | M | MZF |  |  |  |  |  | SE |  |
| dab1485 | ND | UWBM | 11.99 | -86.26 | Nicaragua |  |  | SE |  |
| dab1486 | ND | UWBM | 11.99 | -86.26 | Nicaragua |  |  | SE |  |
| dhb4369 | ND | UWBM | 14.65 | -91.60 | Guatemala |  |  | SE |  |
| 5934 | ND | KU | 13.81 | -89.81 | El Salvador |  |  | SE |  |
| 5937 | ND | KU | 13.81 | -89.81 | El Salvador |  |  | SE |  |
| 5949 | ND | KU | 13.81 | -89.81 | El Salvador |  |  | SE |  |
| 5974 | ND | KU | 13.83 | -89.57 | El Salvador |  |  | SE |  |
| 5978 | ND | KU | 13.83 | -89.57 | El Salvador |  |  | SE |  |
| 5982 | ND | KU | 13.83 | -89.57 | El Salvador |  |  | SE |  |
| 5985 | ND | KU | 13.83 | -89.57 | El Salvador |  |  | SE |  |
| dab1484 | ND | SEC | 11.99 | -86.26 | Nicaragua |  |  | SE |  |
| dab1508 | ND | SEC | 11.99 | -86.26 | Nicaragua |  |  | SE |  |
| dab1349 | ND | SEC | 13.02 | -85.92 | Nicaragua |  |  | SE |  |
| zmuc130351 | ND | SEC | 10.31 | -84.81 | Costa Rica |  |  | SE |  |
| gav2110 | ND | SEC | 15.43 | -86.52 | Honduras |  |  | SE |  |
| gms145 | ND | SEC | 15.43 | -86.52 | Honduras |  |  | SE |  |
| dhb4362 | ND | SEC | 14.65 | -91.60 | Guatemala |  |  | SE |  |
| dhb4377 | ND | SEC | 14.65 | -91.60 | Guatemala |  |  | SE |  |
| dhb4399 | ND | SEC | 14.65 | -91.60 | Guatemala |  |  | SE |  |
| 434136 | ND | SEC | 13.93 | -89.84 | El Salvador |  |  | SE |  |
| 434137 | ND | SEC | 13.68 | -89.66 | El Salvador |  |  | SE |  |
| 229144 | F | SI | 9.14 | -79.72 | Panama |  | x | PA |  |
| 433980 | M | SI | 8.71 | -79.91 | Panama |  | x | PA | GMS1070 |
| 459208 | M | SI | 8.73 | -82.66 | Panama |  | x | PA |  |
| 471747 | F | SI | 8.25 | -81.87 | Panama |  | x | PA | JK04166 |
| 477183 | M | SI | 8.62 | -80.58 | Panama |  | x | PA | GMS1113 |
| 477184 | F | SI | 8.62 | -80.58 | Panama |  | x | PA | GMS1113 |
| 534190 | M | SI | 8.99 | -79.54 | Panama |  | x | PA |  |
| 77897 | F | AMNH | 8.78 | -82.45 | Panama | Chiriqui | x | PA |  |
| 77943 | M | AMNH | 8.54 | -82.58 | Panama | Chiriqui | x | PA |  |
| 136341 | F | AMNH | 8.79 | -80.01 | Panama |  | x | PA | GMS1070 |
| 183029 | F | AMNH | 8.12 | -81.08 | Panama |  |  | PA |  |
| 183032 | M | AMNH | 8.12 | -81.08 | Panama | Veraguas | x | PA | JK160 |
| 183042 | F | AMNH | 8.30 | -81.82 | Panama |  |  | PA |  |
| 186977 | M | AMNH | 7.96 | -80.99 | Panama |  | x | PA | JK04138 |
| 186983 | M | AMNH | 7.96 | -80.99 | Panama |  | x | PA | JK04166 |
| 187939 | F | AMNH | 8.51 | -81.08 | Panama |  | x | PA | JK04138 |
| 187942 | F | AMNH | 8.51 | -81.08 | Panama |  | x | PA | GMS1013 |
| 187946 | F | AMNH | 8.51 | -81.08 | Panama |  | x | PA | ANSP5772 |
| 233529 | F | AMNH | 8.79 | -80.01 | Panama |  | x | PA |  |
| 233530 | F | AMNH | 8.79 | -80.01 | Panama |  | x | PA |  |
| 246532 | M | AMNH | 7.95 | -80.44 | Panama |  | x | PA | ANSP5772 |
| 246533 | M | AMNH | 7.95 | -80.44 | Panama |  | x | PA | ANSP5771 |
| 246534 | F | AMNH | 7.95 | -80.44 | Panama |  | x | PA | ANSP5771 |
| 510442 | M | AMNH | 8.58 | -82.39 | Panama |  |  | PA |  |
| 510444 | F | AMNH | 8.48 | -82.62 | Panama |  |  | PA |  |
| 510445 | M | AMNH | 8.58 | -82.39 | Panama | Chiriqui | x | PA | GMS1013 |
| 510446 | F | AMNH | 8.58 | -82.39 | Panama |  | x | PA | JK160 |
| jtk04160 | ND | UWBM | 8.05 | -81.10 | Panama |  |  | PA |  |
| gms1113 | ND | SEC | 8.62 | -80.10 | Panama |  |  | PA |  |
| gms1013 | ND | SEC | 8.05 | -81.10 | Panama |  |  | PA |  |
| gms1070 | ND | SEC | 8.62 | -80.10 | Panama |  |  | PA |  |
| jtk04138 | ND | SEC | 8.05 | -81.10 | Panama |  |  | PA |  |
| jtk04166 | ND | SEC | 8.05 | -81.10 | Panama |  |  | PA |  |
| ansp5772 | ND | SEC | 8.10 | -80.98 | Panama |  |  | PA |  |
| ansp5771 | ND | SEC | 8.10 | -80.98 | Panama |  |  | PA |  |
| 308390 | M | SI | -17.41 | -63.85 | Bolivia |  | x | WS | 12591 |
| 308391 | F | SI | -17.41 | -63.85 | Bolivia |  | x | WS | 12591 |
| 308392 | M | SI | -6.82 | -66.15 | W Brazil |  | x | WS | ANSP1495 |
| 308393 | F | SI | -4.78 | -56.60 | W Brazil |  | x | WS | ZMUC120394 |
| 327553 | F | SI | 0.56 | -68.14 | W Brazil |  | x | WS | 457560 |
| 138395 | M | AMNH | -17.41 | -66.17 | Bolivia |  | x | WS | 22625 |
| 138407 | F | AMNH | -17.41 | -66.17 | Bolivia |  | x | WS | 12594 |
| 138408 | F | AMNH | -17.41 | -66.17 | Bolivia |  | x | WS | B18345 |
| 138410 | F | AMNH | -17.41 | -66.17 | Bolivia |  | x | WS | 22625 |
| 138413 | M | AMNH | -17.42 | -66.16 | Bolivia |  | x | WS | B1052 |
| 138414 | M | AMNH | -17.42 | -66.16 | Bolivia |  | x | WS | B22623 |
| 138415 | F | AMNH | -17.42 | -66.16 | Bolivia |  | x | WS | ZMUC145305 |
| 147199 | M | AMNH | -3.13 | -60.00 | W Brazil |  | x | WS |  |
| 147200 | F | AMNH | -3.13 | -60.00 | W Brazil |  |  | WS |  |
| 148916 | M | AMNH | -16.95 | -65.38 | Bolivia |  | x | WS | ZMUC145305 |
| 169529 | F | AMNH | -11.06 | -75.33 | Peru |  | x | WS | B11166 |
| 179777 | M | AMNH | -0.75 | -80.37 | Ecuador |  | x | WS |  |
| 179778 | M | AMNH | -0.75 | -80.37 | Ecuador |  | x | WS |  |
| 179779 | F | AMNH | -0.75 | -80.37 | Ecuador |  | x | WS |  |
| 183281 | M | AMNH | -0.70 | -77.1333 | Ecuador |  | x | WS |  |
| 183283 | F | AMNH | -0.70 | -77.1333 | Ecuador |  | x | WS |  |
| 183796 | F | AMNH | -0.70 | -77.1333 | Ecuador |  | x | WS |  |
| 232876 | F |  |  |  |  |  |  | WS |  |
| 235325 | M | AMNH | -6.11 | -77.21 | Peru |  | x | WS | B40061 |
| 235326 | F | AMNH | -6.11 | -77.21 | Peru |  | x | WS | B40060 |
| 239566 | M | AMNH | -9.83 | -73.09 | Peru |  |  | WS |  |
| 239568 | M | AMNH | -9.83 | -73.09 | Peru |  |  | WS | 628 |
| 239569 | F | AMNH | -9.83 | -73.09 | Peru |  | x | WS | 636 |
| 240789 | M | AMNH | -11.81 | -77.17 | Peru |  |  | WS |  |
| 256968 | F | AMNH | -12.05 | -77.03 | Peru |  | x | WS | ANSP1495 |
| 278369 | M |  |  |  |  |  |  | WS |  |
| 278364 | M | AMNH | -10.42 | -65.40 | Bolivia |  | x | WS | 8909 |
| 278367 | F | AMNH | -10.42 | -65.40 | Bolivia |  | x | WS | 8909 |
| 278371 | F | AMNH | -10.42 | -65.40 | Bolivia |  | x | WS | 8959 |
| 278854 | M | AMNH | -10.42 | -65.40 | Bolivia |  | x | WS | 8959 |
| 280309 | M | AMNH | -6.90 | -62.11 | W Brazil |  | x | WS | 457 |
| 282930 | M | AMNH | -6.90 | -62.11 | W Brazil |  | x | WS | ZMUC120394 |
| 288002 | M | AMNH | -4.78 | -56.60 | W Brazil |  | x | WS |  |
| 288012 | F | AMNH | -2.40 | -54.68 | W Brazil |  | x | WS |  |
| 309714 | M | AMNH | -3.35 | -64.71 | W Brazil |  | x | WS | 457558 |
| 309716 | M | AMNH | -3.11 | -43.22 | W Brazil |  | x | WS |  |
| 309719 | F | AMNH | -5.00 | -63.00 | W Brazil |  | x | WS | 457559 |
| 428939 | M | AMNH | -4.78 | -56.60 | W Brazil |  |  | WS |  |
| 428945 | F | AMNH | -2.45 | -54.70 | W Brazil |  |  | WS |  |
| 430092 | F | AMNH | -7.27 | -52.61 | W Brazil |  |  | WS |  |
| 430093 | M | AMNH | -7.27 | -52.61 | W Brazil |  | x | WS |  |
| 435435 | M | AMNH | -0.10 | -67.47 | W Brazil |  | x | WS | 457560 |
| 435436 | M | AMNH | 0.03 | -67.27 | W Brazil |  | x | WS | 457559 |
| 510397 | F | AMNH | -16.33 | -59.62 | Bolivia |  | x | WS | B1052 |
| 510398 | M | AMNH | -16.33 | -59.62 | Bolivia |  |  | WS |  |
| 510399 | M | AMNH | -16.29 | -63.59 | Bolivia |  | x | WS | 12594 |
| 510401 | M | AMNH | -16.93 | -63.63 | Bolivia |  | x | WS | B18345 |
| 510403 | F | AMNH | -3.39 | -64.94 | W Brazil |  | x | WS | 457558 |
| 510404 | F | AMNH | -8.75 | -63.88 | W Brazil |  | x | WS | 36646 |
| 510406 | F | AMNH | -6.90 | -62.11 | W Brazil |  | x | WS |  |
| 510410 | M | AMNH | -6.90 | -62.11 | W Brazil |  | x | WS | 36646 |
| 510411 | M | AMNH | -5.24 | -75.66 | Peru |  | x | WS | 27368 |
| 510415 | F | AMNH | -5.90 | -76.11 | Peru |  | x | WS | 27369 |
| 819679 | M | AMNH | -13.73 | -72.71 | Peru |  | x | WS | 722 |
| 819878 | M | AMNH | -12.15 | -73.23 | Peru |  | x | WS | FMNH398440 |
| 819879 | F | AMNH | -12.15 | -73.23 | Peru |  | x | WS | 711 |
| 22625 | ND | LSU | -16.50 | -68.15 | Bolivia |  |  | WS |  |
| 8909 | ND | LSU | -10.80 | -67.00 | Bolivia |  |  | WS |  |
| 8959 | ND | LSU | -10.80 | -67.00 | Bolivia |  |  | WS |  |
| 12591 | ND | LSU | -17.87 | -63.00 | Bolivia |  |  | WS |  |
| 12594 | ND | LSU | -17.87 | -63.00 | Bolivia |  |  | WS |  |
| 36646 | ND | LSU | -11.51 | -63.58 | W Brazil |  |  | WS |  |
| 27368 | ND | LSU | -4.23 | -74.22 | Peru |  |  | WS |  |
| 27369 | ND | LSU | -4.23 | -74.22 | Peru |  |  | WS |  |
| 457 | ND | KU | -12.33 | -69.03 | Peru |  |  | WS |  |
| 628 | ND | KU | -12.33 | -69.03 | Peru |  |  | WS |  |
| 636 | ND | KU | -12.33 | -69.03 | Peru |  |  | WS |  |
| 711 | ND | KU | -12.33 | -69.03 | Peru |  |  | WS |  |
| 722 | ND | KU | -12.33 | -69.03 | Peru |  |  | WS |  |
| 1052 | ND | SEC | -16.50 | -68.15 | Bolivia |  |  | WS |  |
| 22623 | ND | SEC | -16.50 | -68.15 | Bolivia |  |  | WS |  |
| 18345 | ND | SEC | -17.87 | -63.00 | Bolivia |  |  | WS |  |
| zmuc145305 | ND | SEC | -14.38 | -65.10 | Bolivia |  |  | WS |  |
| zmuc120394 | ND | SEC | -9.45 | -55.86 | W Brazil |  |  | WS |  |
| 457560 | ND | SEC | -1.68 | -65.83 | W Brazil |  |  | WS |  |
| 457559 | ND | SEC | -1.88 | -66.93 | W Brazil |  |  | WS |  |
| 457558 | ND | SEC | -2.49 | -68.26 | W Brazil |  |  | WS |  |
| 40060 | ND | SEC | -4.23 | -74.22 | Peru |  |  | WS |  |
| 40061 | ND | SEC | -4.23 | -74.22 | Peru |  |  | WS |  |
| 11166 | ND | SEC | -9.83 | -73.09 | Peru |  |  | WS |  |
| fmnh398440 | ND | SEC | -12.67 | -71.27 | Peru |  |  | WS |  |
| ANSP 1495 | ND | SEC | -10.00 | -76.00 | Peru |  |  | WS |  |
| 173431 | M | SI | -25.66 | -56.96 | Paraguay |  | x | ES | 205 |
| 368410 | M | SI | -19.56 | -40.44 | E Brasil |  | x | ES |  |
| 515988 | M | SI | -22.93 | -43.24 | E Brasil |  | x | ES |  |
| 128944 | M | AMNH | -23.57 | -46.96 | E Brasil |  | x | ES | ZMUC137118 |
| 146798 | M | AMNH | -25.60 | -54.58 | Argentina |  | x | ES | 313 |
| 154264 | M | AMNH | -25.99 | -54.63 | Argentina |  |  | ES |  |
| 156397 | M | AMNH | -18.92 | -48.31 | E Brazil |  | x | ES |  |
| 245524 | M | AMNH | -6.89 | -38.56 | E Brazil |  | x | ES |  |
| 245525 | F | AMNH | -6.89 | -38.56 | E Brazil |  | x | ES |  |
| 245527 | F | AMNH | -6.89 | -38.56 | E Brazil |  | x | ES |  |
| 245528 | M | AMNH | -13.69 | -40.09 | E Brazil |  | x | ES |  |
| 316394 | M | AMNH | -27.24 | -50.22 | E Brazil |  | x | ES |  |
| 316395 | M | AMNH | -27.24 | -50.22 | E Brazil |  | x | ES |  |
| 316396 | F | AMNH | -27.24 | -50.22 | E Brazil |  | x | ES | B25830 |
| 316401 | M | AMNH | -27.24 | -50.22 | E Brazil |  | x | ES | DHB1801 |
| 316402 | F | AMNH | -27.24 | -50.22 | E Brazil |  |  | ES |  |
| 316403 | F | AMNH | -20.36 | -50.70 | E Brazil |  | x | ES | ZMUC137117 |
| 316406 | M | AMNH | -30.03 | -51.22 | E Brazil |  | x | ES | 3785 |
| 316407 | M | AMNH | -30.03 | -51.22 | E Brazil |  | x | ES | 3852 |
| 316408 | F | AMNH | -30.03 | -51.22 | E Brazil |  |  | ES |  |
| 316409 | F | AMNH | -30.03 | -51.22 | E Brazil |  | x | ES | 3852 |
| 316412 | F | AMNH | -30.03 | -51.22 | E Brazil |  | x | ES | 3853 |
| 316413 | M | AMNH | -30.03 | -51.22 | E Brazil |  | x | ES | 3853 |
| 316414 | M | AMNH | -30.03 | -51.22 | E Brazil |  | x | ES | MVZ168909 |
| 316415 | F | AMNH | -30.03 | -51.22 | E Brazil |  | x | ES | MVZ168909 |
| 318029 | F | AMNH | -19.71 | -40.48 | E Brazil |  | x | ES |  |
| 318030 | M | AMNH | -19.38 | -40.07 | E Brazil |  | x | ES |  |
| 318031 | M | AMNH | -19.38 | -40.07 | E Brazil |  | x | ES |  |
| 318032 | M | AMNH | -19.38 | -40.07 | E Brazil |  | x | ES |  |
| 318033 | M | AMNH | -19.38 | -40.07 | E Brazil |  | x | ES |  |
| 318038 | F | AMNH | -19.71 | -40.48 | E Brazil |  | x | ES |  |
| 318230 | M | AMNH | -19.82 | -43.95 | E Brazil |  | x | ES |  |
| 318231 | F | AMNH | -19.82 | -43.95 | E Brazil |  | x | ES | FMNH345472 |
| 319282 | M | AMNH | -23.89 | -55.43 | E Brazil |  | x | ES | ZMUC144784 |
| 320262 | M | AMNH | -25.69 | -56.26 | Paraguay |  | x | ES | B25909 |
| 320711 | M | AMNH | -23.44 | -58.44 | Paraguay |  | x | ES |  |
| 320713 | F | AMNH | -23.44 | -58.44 | Paraguay |  | x | ES | 3785 |
| 320714 | M | AMNH | -25.26 | -57.56 | Paraguay |  | x | ES | 25853 |
| 510390 | M | AMNH | -23.55 | -46.63 | E Brazil |  | x | ES | ZMUC137117 |
| 510391 | M | AMNH | -23.55 | -46.63 | E Brazil |  | x | ES | FMNH345472 |
| 510393 | M | AMNH | -23.55 | -46.63 | E Brazil |  | x | ES |  |
| 510395 | F | AMNH | -23.55 | -46.63 | E Brazil |  | x | ES | ZMUC137118 |
| 774270 | M | AMNH | -25.90 | -54.61 | Argentina |  | x | ES | GAV821 |
| 774272 | M | AMNH | -25.90 | -54.61 | Argentina |  | x | ES | GAV822 |
| 774276 | F | AMNH | -25.90 | -54.61 | Argentina |  | x | ES | GAV821 |
| 774277 | F | AMNH | -25.90 | -54.61 | Argentina |  | x | ES | GAV822 |
| 774278 | F | AMNH | -25.90 | -54.61 | Argentina |  | x | ES | 226 |
| 774280 | F | AMNH | -25.90 | -54.61 | Argentina |  | x | ES | 259 |
| 774281 | F | AMNH | -25.90 | -54.61 | Argentina |  | x | ES | DHB1801 |
| 813091 | F | AMNH | -29.44 | -49.80 | E Brazil |  | x | ES | 3662 |
| dhb1801 | ND | UWBM | -26.96 | -55.09 | Argentina |  |  | ES |  |
| gav821 | ND | UWBM | -26.96 | -55.09 | Argentina |  |  | ES |  |
| gav822 | ND | UWBM | -26.96 | -55.09 | Argentina |  |  | ES |  |
| 25853 | ND | LSU | -26.23 | -56.02 | Paraguay |  |  | ES |  |
| 205 | ND | KU | -26.35 | -55.52 | Paraguay |  |  | ES |  |
| 226 | ND | KU | -26.35 | -55.52 | Paraguay |  |  | ES |  |
| 259 | ND | KU | -26.35 | -55.52 | Paraguay |  |  | ES |  |
| 313 | ND | KU | -26.35 | -55.52 | Paraguay |  |  | ES |  |
| 3662 | ND | KU | -26.52 | -55.80 | Paraguay |  |  | ES |  |
| 3785 | ND | KU | -26.52 | -55.80 | Paraguay |  |  | ES |  |
| 3852 | ND | KU | -26.52 | -55.80 | Paraguay |  |  | ES |  |
| 3853 | ND | KU | -26.52 | -55.80 | Paraguay |  |  | ES |  |
| ic1110 | ND | SEC | 8.31 | -70.05 | Venezuela |  |  | ES |  |
| zmuc137118 | ND | SEC | -23.61 | -46.46 | E Brazil |  |  | ES |  |
| zmuc137117 | ND | SEC | -23.61 | -46.46 | E Brazil |  |  | ES |  |
| fmnh345472 | ND | SEC | -23.61 | -46.46 | E Brazil |  |  | ES |  |
| 25909 | ND | SEC | -25.46 | -56.02 | Paraguay |  |  | ES |  |
| 25830 | ND | SEC | -26.23 | -56.02 | Paraguay |  |  | ES |  |
| zmuc144784 | ND | SEC | -26.07 | -55.75 | Paraguay |  |  | ES |  |
| mvz168909 | ND | SEC | -27.18 | -55.78 | Paraguay |  |  | ES |  |
| 176576 | M |  |  |  |  |  |  | ES |  |

**APPENDIX S2**

Supporting information for method of this study

**Figure S2.1** Tetrahedral color space plots occupied by nine corporal patches measured in 339 males and females of the species *Habia rubica*. This sampling is divided by recently described phylogroups (complete sampling). These phylogroups were named as follows: NP, northern pacific of Mexico; SP, southern pacific of Mexico; GM, Gulf of Mexico; SE, southeastern

**Males**

**
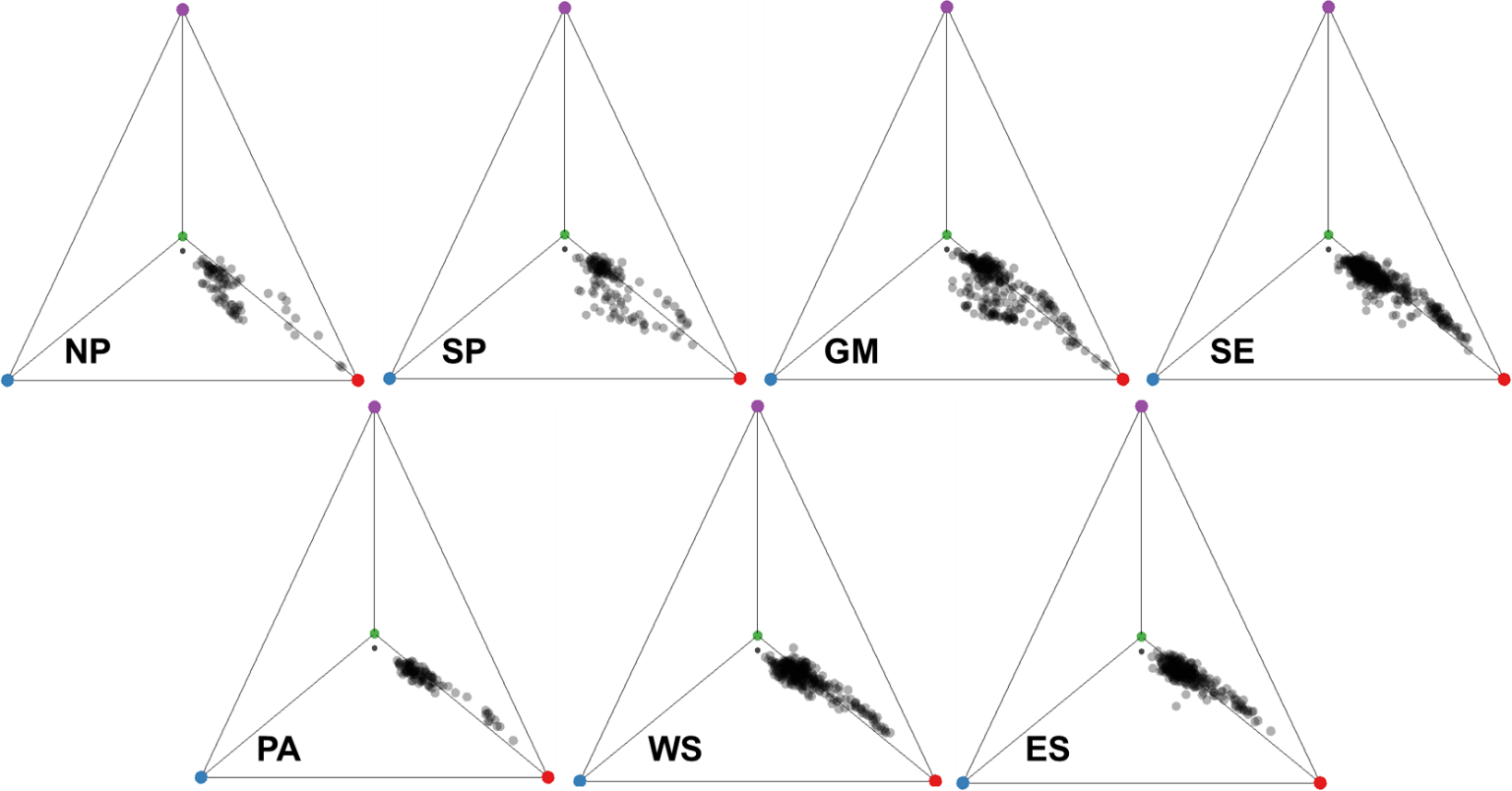
**

**Females**

**
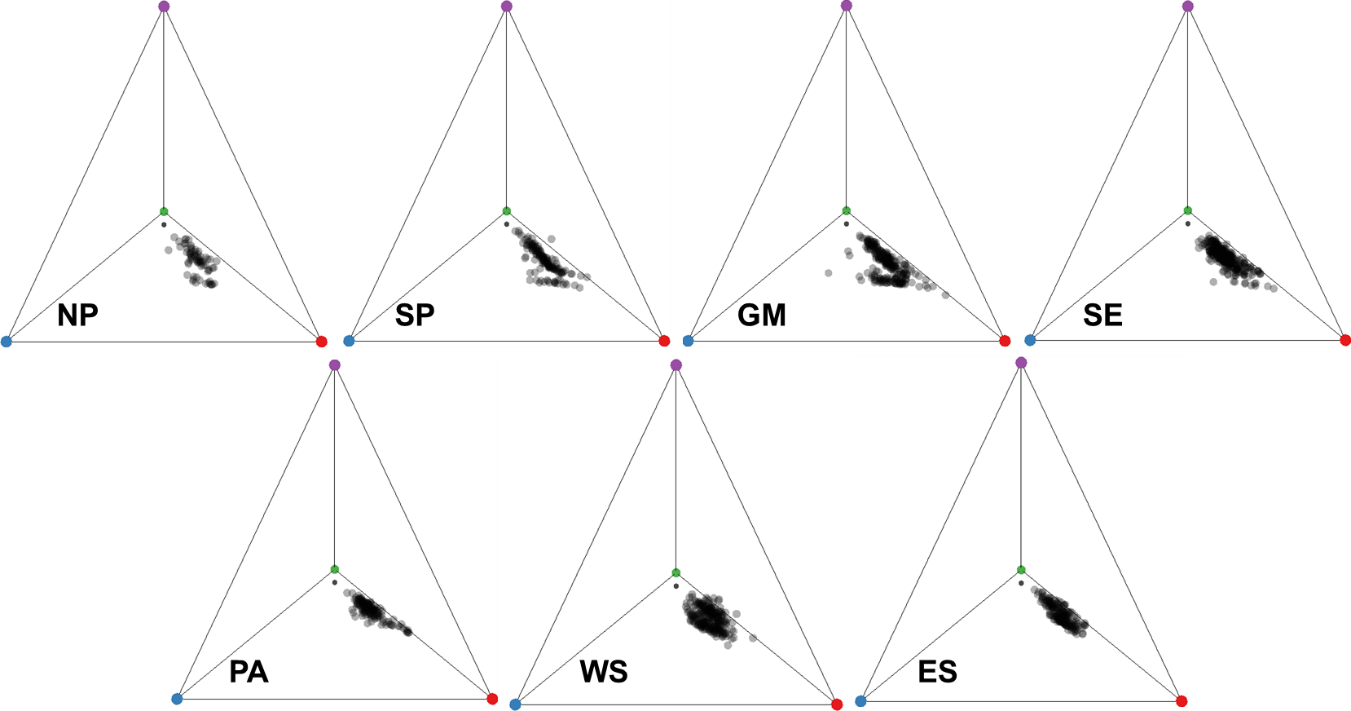
**

**Figure S2.2** (a) Map showing the position of the polygons in raster format for the seven phylogroups that make up the *Habia rubica* sampling: NP, northern pacific of Mexico; SP, southern pacific of Mexico; GM, Gulf of Mexico; SE, southeastern Mexico. (b) Map showing the position of some of the delimited phylogroups from individuals with genetic and phenotypic sampling: M_NP, males from northern pacific of Mexico; M_SP, males from southern pacific of Mexico; F_GM, females from Gulf of Mexico; M_SE, males from southeastern Mexico.

**
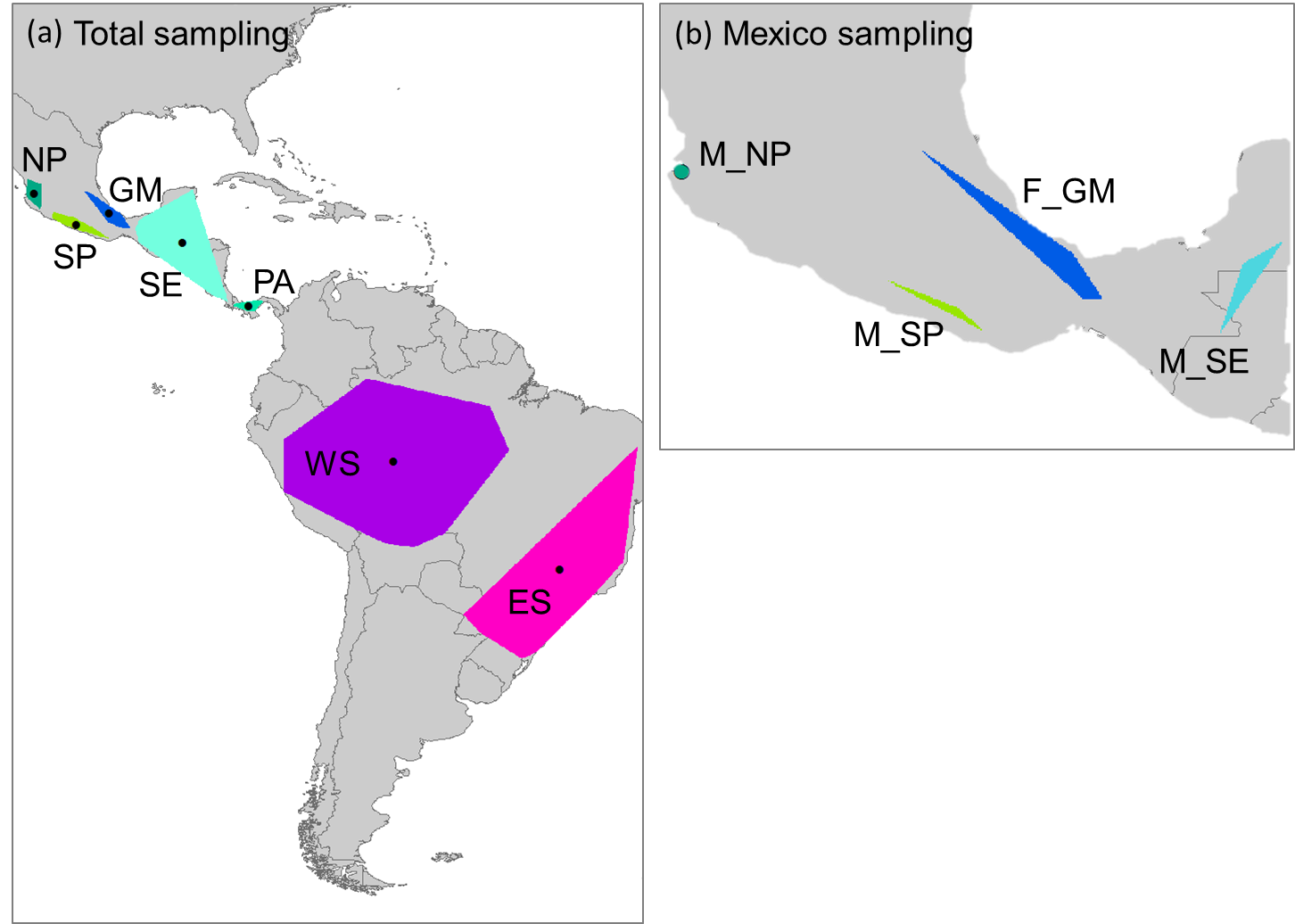
**

**Figure S2.3** Comparison graph between mean and median values ​​of 19 bioclimatic variables obtained for raster cell coordinates for each polygon (phylogroup) of the complete distribution of *Habia rubica*.

**
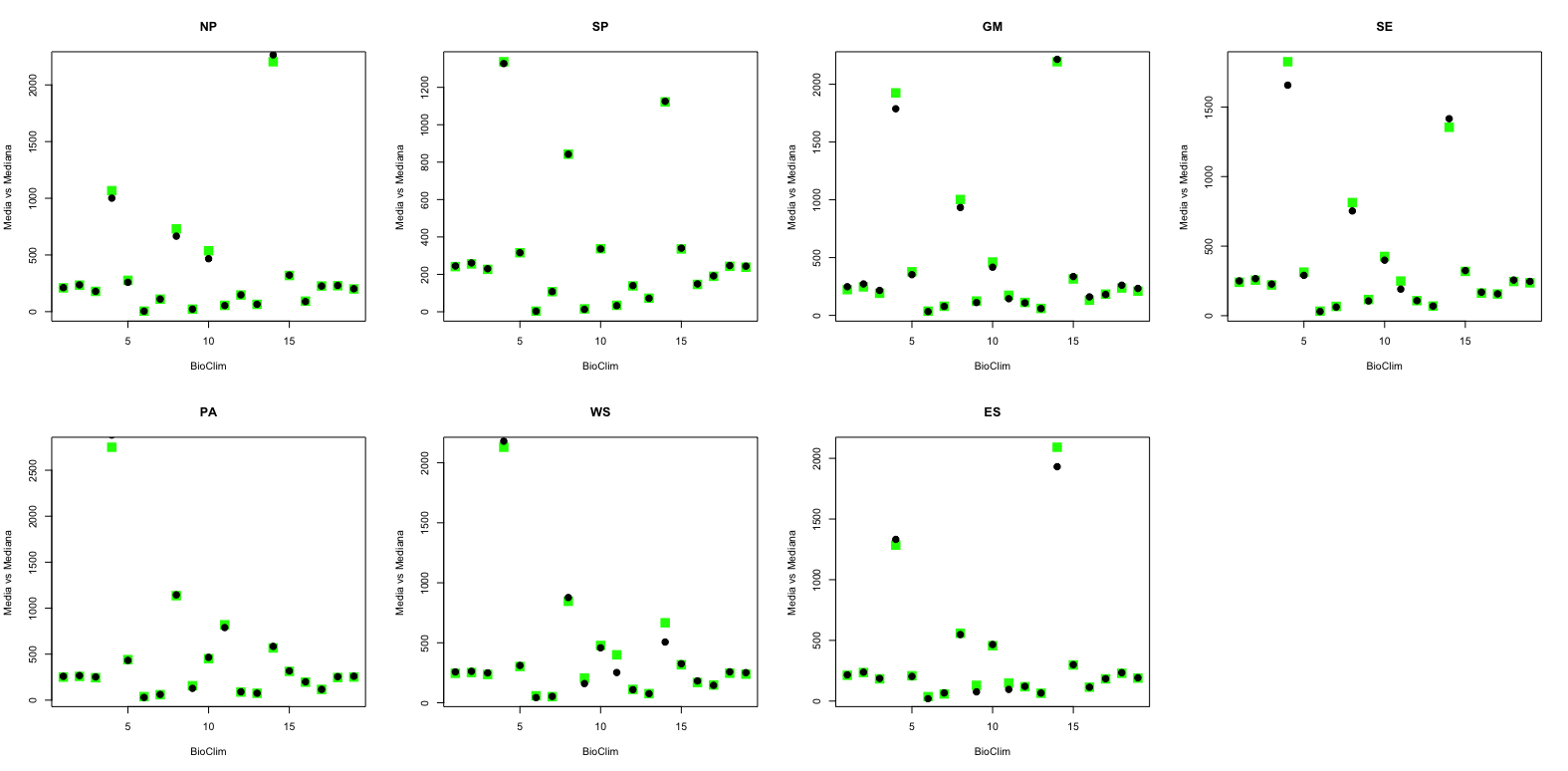
**

**Figure S2.4** Comparison graph between mean and median values ​​of 19 bioclimatic variables obtained for raster cell coordinates for each polygon (phylogroup) of the distribution in Mexico of *Habia rubica*.

**
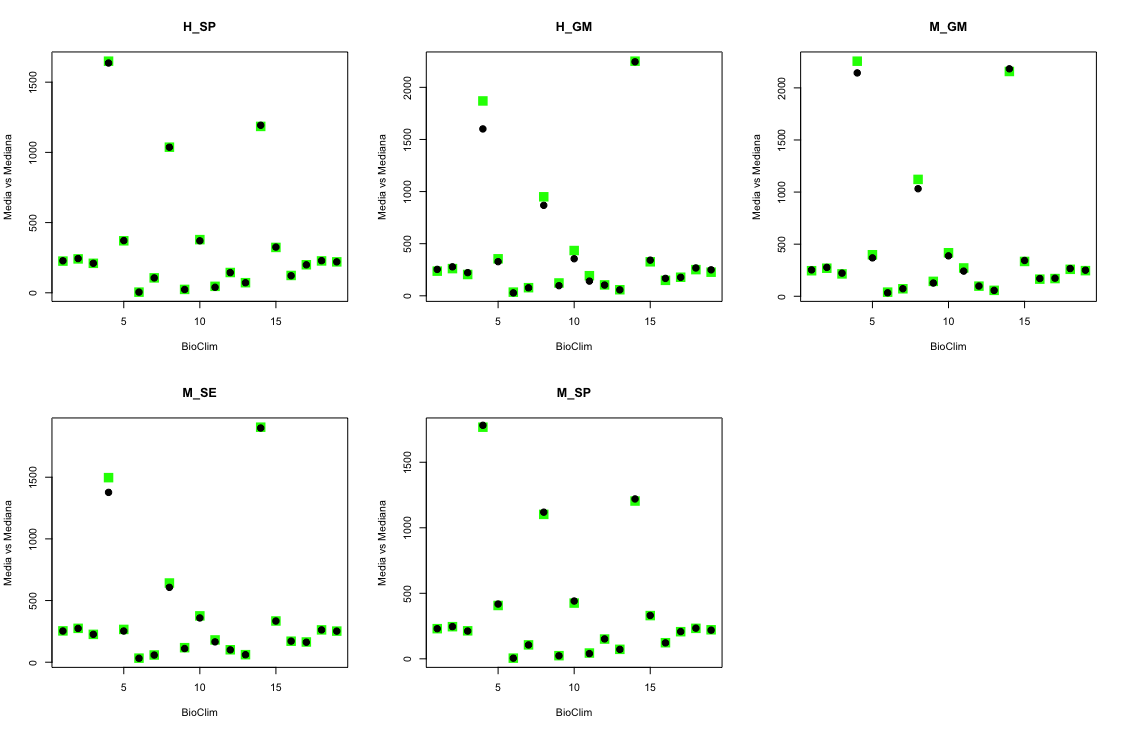
**

**Table S2.1** Test for to sexual dimorphism for three morphometric measurements of individuals from *Habia rubica* populations.

|  | n  (males) | n  (females) | Males mean ± sd | Females  mean ± sd | Mean  difference | 95% CI  Lower | 95% CI  Upper | *t* | df | *p-*value |
| --- | --- | --- | --- | --- | --- | --- | --- | --- | --- | --- |
| Wing | 110 | 104 | 90.35 ± 4.07 | 84.04 ± 4.32 | 6.31 | -7.44 | -5.17 | -10.97 | 209.21 | <0.05 |
| Tarsus | 110 | 104 | 23.73 ± 1.32 | 23.20 ± 1.47 | 0.53 | -0.91 | -0.16 | -2.80 | 206.63 | <0.05 |
| Tail | 110 | 104 | 81.39 ± 5.16 | 75.91 ± 5.17 | 5.48 | -6.87 | -4.08 | -7.75 | 211.28 | <0.05 |
| H0: Difference in means = 0 | | | | | | | | | | |

**Table S2.2** Bioclimatic variables obtained from WorldClim. The bioclimatic variables represent annual trends, seasonality and extreme or limiting environmental factors. A quarter is a period of three months (1/4 of the year).

| Name of layer | Varibles |
| --- | --- |
| BIOO1 | Annual Mean Temperature |
| BIOO2 | Mean Diurnal Range (Mean of monthly (max temp - min temp)) |
| BIOO3 | Isothermality (BIO2/BIO7) (* 100) |
| BIOO4 | Temperature Seasonality (standard deviation *100) |
| BIOO5 | Max Temperature of Warmest Month |
| BIOO6 | Min Temperature of Coldest Month |
| BIOO7 | Temperature Annual Range (BIO5-BIO6) |
| BIOO8 | Mean Temperature of Wettest Quarter |
| BIOO9 | Mean Temperature of Driest Quarter |
| BIO10 | Mean Temperature of Warmest Quarter |
| BIO11 | Mean Temperature of Coldest Quarter |
| BIO12 | Annual Precipitation |
| BIO13 | Precipitation of Wettest Month |
| BIO14 | Precipitation of Driest Month |
| BIO15 | Precipitation Seasonality (Coefficient of Variation) |
| BIO16 | Precipitation of Wettest Quarter |
| BIO17 | Precipitation of Driest Quarter |
| BIO18 | Precipitation of Warmest Quarter |
| BIO19 | Precipitation of Coldest Quarter |

**Appendix S3**

Supporting information for results of this study

**Table S3.1** Paired corrected genetic distances (percentage of differentiation), estimated using the nucleotide model Jukes-Cantor (Jukes & Cantor, 1969). The names of rows and columns correspond to the names of each identified group within the species had rubica (Ramírez-Barrera et al., 2018).

|  | NP | SP | GM | SE | PA | WS | ES |
| --- | --- | --- | --- | --- | --- | --- | --- |
| NP |  |  |  |  |  |  |  |
| SP | 1.0 |  |  |  |  |  |  |
| GM | 5.1 | 4.4 |  |  |  |  |  |
| SE | 5.1 | 4.5 | 1.7 |  |  |  |  |
| PA | 6.1 | 5.5 | 2.7 | 2.4 |  |  |  |
| WS | 7.4 | 7.4 | 5.7 | 5.8 | 6.6 |  |  |
| ES | 6.8 | 7.6 | 5.7 | 5.8 | 6.7 | 6.4 |  |

**Table S3.2** Correlation values estimated between three morphometric variables (wing length, tarsus length, tail length) obtained from individuals of *Habia rubica*.

| Females | | | |
| --- | --- | --- | --- |
|  | Wing length | Tarsus length | Tail length |
| Wing length | 1.00 | 0.21 | 0.49 |
| Tarsus length | 0.21 | 1.00 | 0.65 |
| Tail length | 0.49 | 0.65 | 1.00 |
| Males | | | |
| Wing length | 1.00 | 0.32 | 0.56 |
| Tarsus length | 0.32 | 1.00 | 0.57 |
| Tail length | 0.56 | 0.57 | 1.00 |

**Figure S3.2** Graphs of correlation between three morphometric variables (wing length, tarsus length, tail length) obtained from males of *Habia rubica*.
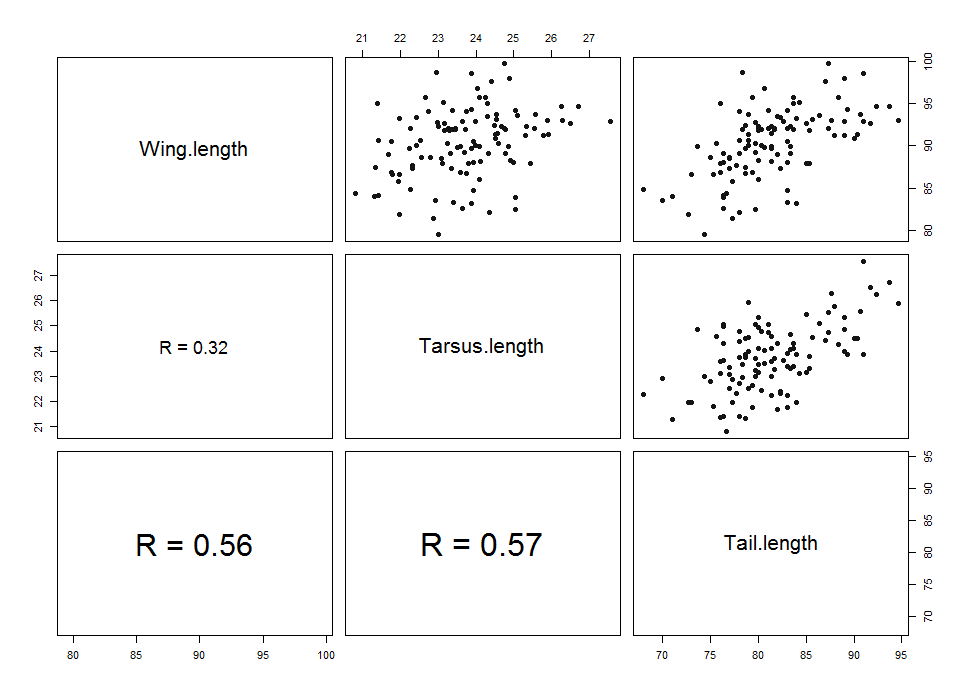


**Figure S3.3** Correlation graphs between three morphometric variables (wing length, tarsal length, tail length) obtained from the female individuals of *Habia rubica*.

**
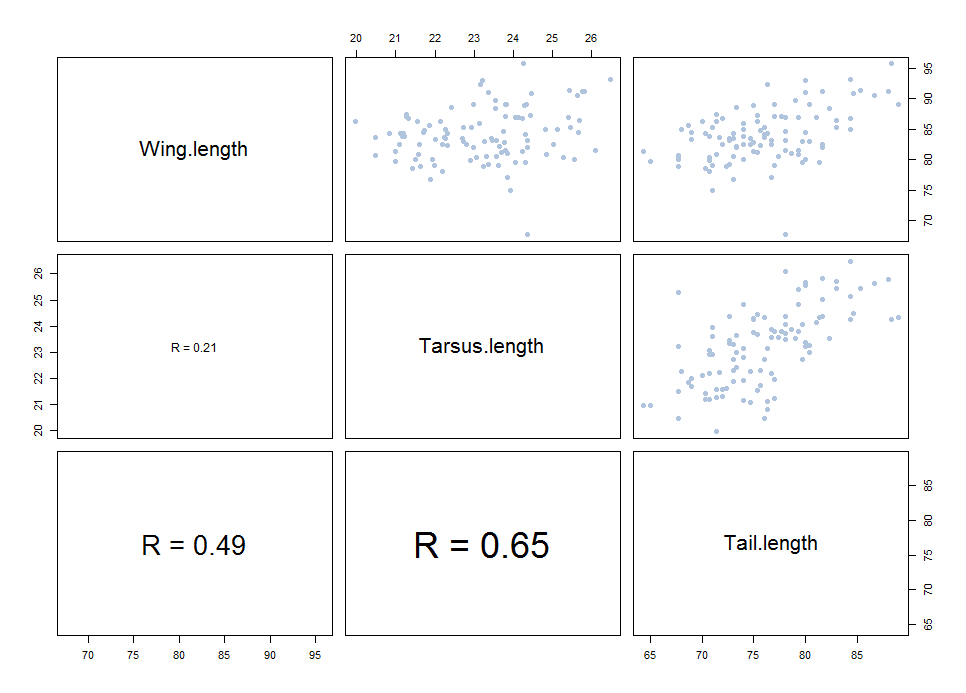
**

**Table S3.3** Results of the PCA analysis carried out to summarize morphometric variables used as estimation of body size of males and females of the species *Habia rubica* in subsequent analyzes. The data were grouped by phylogroups according to Ramírez-Barrera et al. (2018).

| PHYLOGROUPS MALES | PC1 | PC2 | PC3 |
| --- | --- | --- | --- |
| Wing length | 0.4194101 | 0.9074694 | 0.02438163 |
| Tarsus length | 0.644541 | -0.2787628 | -0.71193977 |
| Tail length | 0.6392669 | -0.3143097 | 0.70181714 |
| PHYLOGROUPS FEMALES |  |  |  |
| Wing length | 0.4540808 | -0.8909452 | -0.00522271 |
| Tarsus length | 0.6294597 | 0.3249492 | -0.70582473 |
| Tail length | 0.6305483 | 0.317214 | 0.70836726 |

| PHYLOGROUPS MALES | PC1 | PC2 | PC3 |
| --- | --- | --- | --- |
| Standard deviation | 1.4826 | 0.863 | 0.23894 |
| Proportion of Variance | 0.7327 | 0.2482 | 0.01903 |
| Cumulative Proportion | 0.7327 | 0.981 | 1 |
| PHYLOGROUPS FEMALES |  |  |  |
| Standard deviation | 1.5037 | 0.82 | 0.25776 |
| Proportion of Variance | 0.7537 | 0.2241 | 0.02215 |
| Cumulative Proportion | 0.7537 | 0.9778 | 1 |

**Table S3.4** Results of the PCA analysis carried out to summarize morphometric variables used as estimation of body size of males and females of the species *Habia rubica* in subsequent analyzes. This analysis was done by taking the individual data.

| MALES | PC1 | PC2 | PC3 |
| --- | --- | --- | --- |
| Wing length | 0.544 | -0.715 | 0.437 |
| Tarsus length | 0.548 | 0.698 | 0.459 |
| Tail length | 0.634 | 0.009 | -0.772 |
| FEMALES |  |  |  |
| Wing length | 0.481 | -0.809 | 0.335 |
| Tarsus length | 0.576 | 0.580 | 0.575 |
| Tail length | 0.660 | 0.083 | -0.746 |

| MALES |  |  |  |
| --- | --- | --- | --- |
| Standard deviation | 1.406 | 0.825 | 0.583 |
| Proportion of Variance | 0.659 | 0.227 | 0.113 |
| Cumulative Proportion | 0.659 | 0.886 | 1 |
| FEMALES |  |  |  |
| Standard deviation | 1.387 | 0.893 | 0.531 |
| Proportion of Variance | 0.640 | 0.265 | 0.094 |
| Cumulative Proportion | 0.640 | 0.906 | 1 |

**Figure S3.4** Plots of the analysis of PCA made with the total individual data of males and females of the species *Habia rubica*.


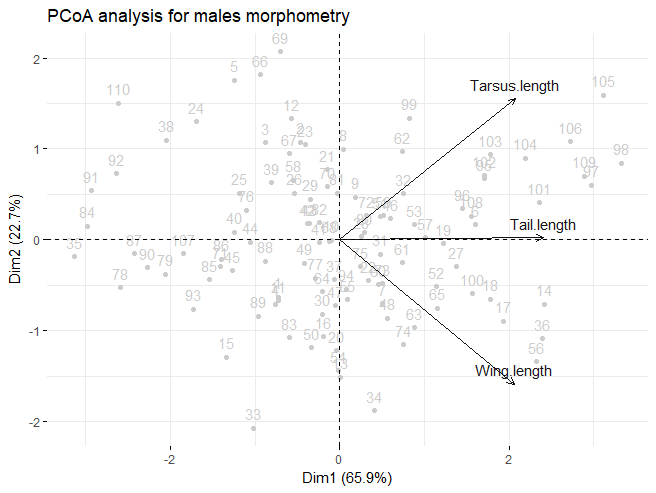

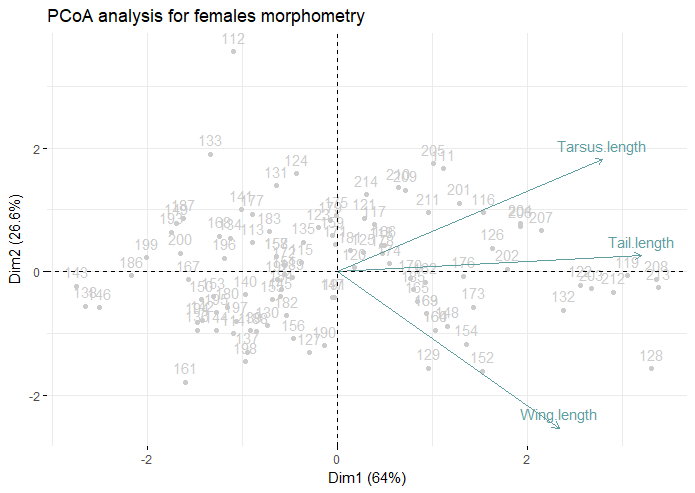


**Table S3.5** Results of the linear regression analysis carried out taking the values of coloration of the plumage (variable hue) and body size (PC1) of males and females of the species *Habia rubica*.

| MALES | Estimate | Std. Error | t value | p value | R^2^ |
| --- | --- | --- | --- | --- | --- |
| Plumaje coloration | 0.020 | 0.005 | 3.582 | <0.05 | 0.098 |
| Body size | -0.015 | 0.006 | -2.628 | <0.05 | 0.05 |
| FEMALES |  |  |  |  |  |
| Plumaje coloration | 0.016 | 0.006 | 2.836 | <0.05 | 0.06 |
| Body size | -0.017 | 0.006 | 0.006 | <0.05 | 0.07 |

**Figure S3.5** Plots of plumage coloration values (variable hue) and body size (PC1) against latitude. Dashed line for females and solid line for males of *Habia rubica* species.

**
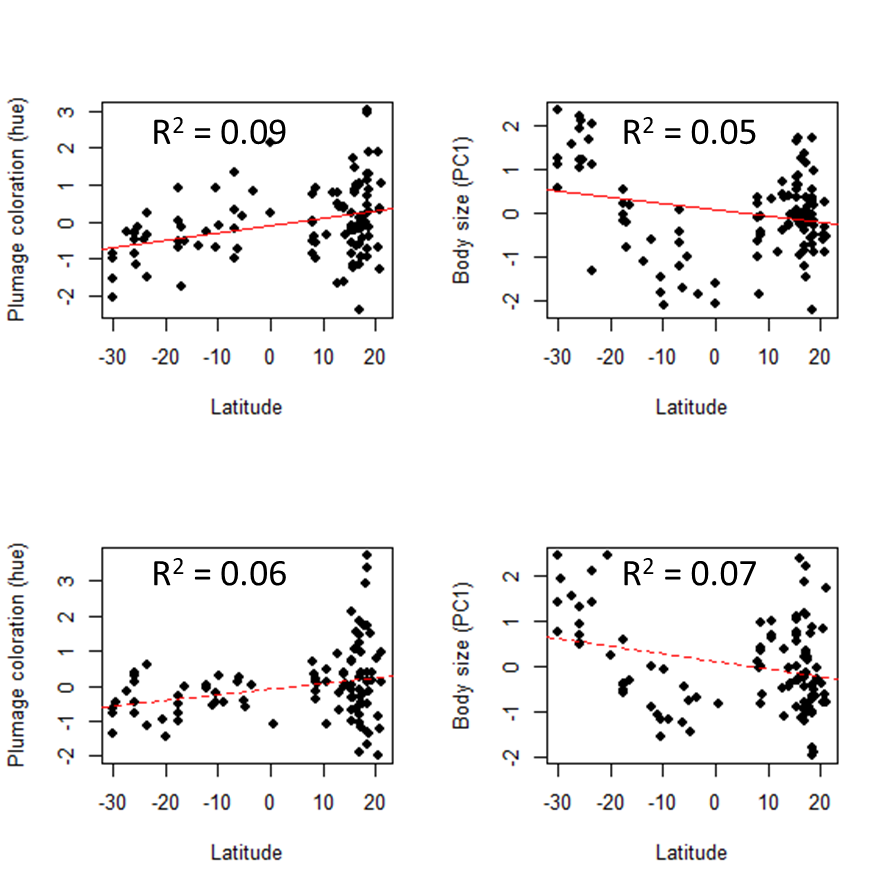
**

**Table S3.6** Results of univariate MMRR analysis grouping by sex for analysis between phylogroups and individuals of distribution in Mexico of *H. rubica,* testing three independent variables of distance: genetics, color and body size. Here, we show the results of coefficient of determination (R^2^), beta-weights (*β*) and *p*-value (*p*) for each predictor.

|  | **Analysis by individuals** | | | | | | **Analysis by phylogroups** | | | | | | |
| --- | --- | --- | --- | --- | --- | --- | --- | --- | --- | --- | --- | --- | --- |
|  | Males | | | Females | | | Males | | | | Females | | |
| a) MRM (**mtDNA** ~ Predictor) | | | | | | | | | | | | | |
|  | R^2^ | *β* | *p* | R^2^ | *β* | *p* | R^2^ | *β* | *p* | R^2^ | | *β* | *p* |
| Geography | **0.31** | **0.50** | **<0.01** | **0.48** | **0.86** | **<0.01** | 0.36 | 0.79 | 0.04 | 0.17 | | 0.54 | 0.17 |
| Climate | **0.34** | **0.18** | **<0.01** | **0.23** | **0.22** | **<0.01** | 0.90 | 0.51 | 0.09 | 0.53 | | 0.41 | 0.16 |
| Hue total | 0.10 | 0.28 | **<0.01** | 0.00 | 0.05 | 0.63 | 0.63 | 0.79 | 0.24 | 0.06 | | -0.26 | 1.00 |
| Chroma total | 0.00 | -0.03 | 0.57 | 0.00 | -0.01 | 0.88 | 0.01 | -0.06 | 0.53 | 0.01 | | -0.10 | 0.15 |
| Volume total | 0.03 | 0.16 | 0.03 | 5.5E-05 | 0.01 | 0.94 | 0.73 | 0.81 | 0.20 | 0.14 | | -0.43 | 0.84 |
| Body size | **0.10** | **0.29** | **0.01** | 0.03 | 0.21 | 0.05 | 0.15 | -0.44 | 0.75 | 0.44 | | 0.66 | 0.17 |
| b) MRM (**Hue** ~ Predictor) | | | | | | | | | | | | | |
|  | R^2^ | *β* | *p* | R^2^ | *β* | *p* | R^2^ | *β* | *p* | R^2^ | | *β* | *p* |
| Geography | 0.013 | 0.11 | 0.32 | 0.05 | 0.20 | 0.06 | 0.09 | 0.40 | 0.44 | 0.28 | | 0.70 | 0.29 |
| Climate | 0.01 | 0.03 | 0.52 | 0.01 | -0.03 | 0.53 | 0.85 | 0.50 | 0.03 | 0.09 | | 0.17 | 0.58 |
| mtDNA | 0.10 | 0.36 | 0.01 | 0.00 | 0.03 | 0.67 | 0.63 | 0.80 | 0.24 | 0.06 | | -0.26 | 1.00 |
| Body size | 0.21 | 0.47 | **<0.01** | 0.01 | -0.08 | 0.56 | 0.04 | -0.23 | 0.75 | 0.17 | | 0.42 | 0.48 |
| c) MRM (**Chroma** ~ Predictor) | | | | | | | | | | | | | |
| Geography | 3.0E-06 | 0.00 | 0.99 | 0.01 | -0.10 | 0.38 | 0.13 | 0.60 | 0.65 | 0.08 | | -0.40 | 0.63 |
| Climate | 0.02 | -0.05 | 0.37 | 0.01 | -0.04 | 0.45 | 0.05 | -0.15 | 0.27 | 0.00 | | -0.04 | 0.95 |
| mtDNA | 0.00 | -0.04 | 0.60 | 0.00 | -0.01 | 0.91 | 0.01 | -0.10 | 0.57 | 0.01 | | -0.11 | 0.15 |
| Body size | 0.00 | -0.03 | 0.86 | 0.00 | -0.05 | 0.77 | 0.11 | 0.48 | 0.56 | 0.12 | | -0.37 | 0.70 |
| d) MRM (**Volume** ~ Predictor) | | | | | | | | | | | | | |
| Geography | 0.01 | 0.09 | 0.42 | 0.00 | -0.04 | 0.72 | 0.35 | 0.82 | 0.24 | 0.07 | | 0.29 | 0.43 |
| Climate | 0.00 | 0.02 | 0.70 | 0.01 | -0.03 | 0.53 | 0.56 | 0.42 | 0.27 | 0.05 | | 0.11 | 0.67 |
| mtDNA | 0.03 | 0.20 | 0.04 | 5.5E-05 | 0.00 | 0.92 | 0.73 | 0.89 | 0.20 | 0.14 | | -0.31 | 0.82 |
| Body size | 0.02 | 0.13 | 0.38 | 0.01 | -0.08 | 0.50 | 0.03 | -0.22 | 0.96 | 0.03 | | 0.15 | 0.61 |
| e) MRM (**Body size** ~ Predictor) | | | | | | | | | | | | | |
|  | R^2^ | *β* | *p* | R^2^ | *β* | *p* | R^2^ | *β* | *p* | R^2^ | | *β* | *p* |
| Geography | 0.03 | 0.16 | 0.07 | 0.04 | 0.20 | 0.07 | 0.01 | -0.09 | 0.85 | 0.69 | | 1.09 | 0.08 |
| Climate | 0.05 | 0.08 | 0.08 | 0.00 | 0.01 | 0.85 | 0.12 | -0.17 | 0.84 | 0.91 | | 0.53 | 0.03 |
| mtDNA | 0.10 | 0.36 | 0.01 | 0.03 | 0.13 | 0.05 | 0.15 | -0.34 | 0.75 | 0.44 | | 0.65 | 0.17 |
| Hue total | 0.21 | 0.45 | **<0.01** | 0.01 | -0.09 | 0.57 | 0.04 | -0.18 | 0.74 | 0.17 | | 0.41 | 0.44 |
| Chroma total | 0.00 | -0.03 | 0.84 | 0.00 | -0.04 | 0.77 | 0.11 | 0.22 | 0.57 | 0.12 | | -0.32 | 0.70 |
| Volume total | 0.02 | 0.12 | 0.37 | 0.01 | -0.09 | 0.52 | 0.03 | -0.16 | 0.96 | 0.03 | | 0.20 | 0.61 |

**Table S3.7** Results of multivariate MMRR analysis grouped by sex for analysis between phylogroups and individuals of distribution in Mexico of *H. rubica*. We show the results of coefficient of determination (R^2^) Beta-weights (*β*) and *p*-value (*p*) and of each predictor from the overall model.

|  | **Analysis by individuals** | | | | | | **Analysis by phylogroups** | | | | | |
| --- | --- | --- | --- | --- | --- | --- | --- | --- | --- | --- | --- | --- |
|  | Males | | | Females | | | Males | | | Females | | |
| a) MRM (**mtDNA** ~ Geography + Clime + Hue + Body size) | | | | | | | | | | | | |
|  | R^2^ | *β* | *p* | R^2^ | *Β* | *p* | R^2^ | *β* | *p* | R^2^ | *β* | *p* |
| Geography | 0.52 | 0.32 | <0.01 | 0.52 | 0.76 | <0.01 | 0.96 | -0.38 | 0.69 | 0.79 | -0.14 | 0.50 |
| Climate |  | 0.12 | <0.01 |  | 0.09 | <0.01 |  | 1.19 | 0.39 |  | 0.27 | 1.00 |
| Hue |  | 0.18 | 0.01 |  | -0.10 | 0.17 |  | -1.12 | 0.58 |  | -0.56 | 0.67 |
| Body size |  | 0.08 | 0.16 |  | 0.03 | 0.63 |  | 0.16 | 0.77 |  | 0.52 | 0.83 |
| b) MRM (**Hue** ~ Geography + Clime + mtDNA + Body size) | | | | | | | | | | | | |
|  | R^2^ | *β* | *p* | R^2^ | *Β* | *p* | R^2^ | *β* | *p* | R^2^ | *β* | *p* |
| Geography | 0.26 | -0.06 | 0.75 | 0.12 | 0.41 | 0.01 | 0.98 | -0.40 | 0.46 | 0.68 | 0.31 | 0.87 |
| Climate |  | -0.06 | 0.31 |  | -0.07 | 0.23 |  | 0.89 | 0.21 |  | -0.07 | 1.00 |
| mtDNA |  | 0.36 | <0.01 |  | -0.10 | 0.30 |  | -0.51 | 0.38 |  | -0.86 | 0.54 |
| Body size |  | 0.41 | <0.01 |  | -0.14 | 0.27 |  | 0.17 | 0.64 |  | 0.92 | 0.65 |
| c) MRM (**Chroma** ~ Geography + Clime + mtDNA + Body size) | | | | | | | | | | | | |
| Geography | 0.02 | 0.06 | 0.71 | 0.028 | -0.14 | 0.43 | 0.51 | 0.94 | 0.79 | 0.90 | -0.32 | 0.87 |
| Climate |  | -0.06 | 0.40 |  | -0.05 | 0.56 |  | -0.99 | 0.67 |  | 1.92 | 0.41 |
| mtDNA |  | 0.03 | 0.81 |  | 0.12 | 0.28 |  | 1.34 | 0.89 |  | -0.41 | 0.70 |
| Body size |  | -0.01 | 0.94 |  | -0.04 | 0.85 |  | 0.39 | 1.00 |  | -3.19 | 0.31 |
| d) MRM (**Volume** ~ Geography + Clime + mtDNA + Body size) | | | | | | | | | | | | |
| Geography | 0.04 | 0.00 | 0.99 | 0.02 | -0.05 | 0.79 | 0.79 | 0.12 | 0.96 | 0.72 | -0.23 | 0.83 |
| Climate |  | -0.03 | 0.66 |  | -0.04 | 0.54 |  | -0.35 | 0.79 |  | 0.93 | 0.45 |
| mtDNA |  | 0.22 | 0.13 |  | 0.09 | 0.40 |  | 1.52 | 0.49 |  | -1.06 | 0.32 |
| Body size |  | 0.08 | 0.63 |  | -0.09 | 0.51 |  | 0.19 | 1.00 |  | -0.59 | 0.74 |
| e) MRM (**Body size** ~ Geography + Clime + mtDNA + Hue) | | | | | | | | | | | | |
|  | R^2^ | *β* | *p* | R^2^ | *Β* | *p* | R^2^ | *β* | *p* | R^2^ | *β* | *P* |
| Geography | 0.25 | -0.00 | 0.98 | 0.07 | 0.25 | 0.15 | 0.61 | 1.32 | 0.43 | 0.93 | 0.16 | 0.95 |
| Climate |  | 0.04 | 0.45 |  | -0.05 | 0.43 |  | -2.64 | 0.39 |  | 0.38 | 0.57 |
| mtDNA |  | 0.15 | 0.28 |  | 0.04 | 0.71 |  | 1.27 | 0.73 |  | 0.16 | 0.82 |
| Hue |  | 0.40 | <0.01 |  | -0.16 | 0.23 |  | 2.98 | 0.38 |  | 0.18 | 0.61 |
